# The effect of exercise training modalities on the morphological and mechanical properties of the achilles tendon: a systematic review and network meta-analysis

**DOI:** 10.3389/fspor.2026.1782503

**Published:** 2026-03-12

**Authors:** Baisheng Fu, Yihan Qian, Yuan Wang, Junjie Fang, Yaodong Gu, Xini Zhang

**Affiliations:** 1 Faculty of Sport Science, Ningbo University, Ningbo, China; 2 HangZhou Ziyun Experimental School, Hangzhou, China; 3 Radiology Department, Ningbo No.2 Hospital, Ningbo, China; 4 Research Academy of Grand Health, Ningbo University, Ningbo, China

**Keywords:** achilles tendon, gait retraining, network meta-analysis, plyometric exercise, resistance training, tendon stiffness

## Abstract

**Introduction:**

The Achilles tendon (AT) is vital for sports performance yet highly susceptible to injury. Exercise can induce structural and mechanical adaptations, but the relative effectiveness of different protocols remains uncertain. This systematic review and network meta-analysis evaluated exercise-based interventions targeting AT morphology and mechanics in healthy adults.

**Methods:**

PubMed, Web of Science and Scopus were searched from inception to 18 Feb 2025. Randomized controlled trials (RCTs) were synthesized using random-effects network meta-analysis (Hedges' g). Risk of bias was assessed using a modified Downs and Black Quality Index.

**Results:**

Forty-nine trials were included in the systematic review; 31 RCTs (*n* = 1,388) contributed to the network across five intervention categories (15 subtypes). Short-term plyometric jump training ranked highest for increasing AT cross-sectional area (SUCRA 95.6%; SMD 1.33 vs. control). Long-term isometric plantar-flexion training ranked highest for improving stiffness (SUCRA 89.3%). Gait retraining ranked highest for AT length, elongation and strain (SUCRA 59.6%–77.1%), although networks for several outcomes were sparse and estimates imprecise. Stretching-focused programs generally ranked low across outcomes.

**Discussion:**

In healthy adults, progressive, higher-dose loading, particularly isometric plantar-flexion training and plyometric jump training, shows the most favorable rankings for AT adaptation. Rankings should be interpreted cautiously given heterogeneity and sparse networks for some outcomes; well-powered head-to-head RCTs with harmonised outcome definitions are needed.

**Systematic Review Registration:**

https://www.crd.york.ac.uk/prospero, PROSPERO CRD420251009672.

## 1 Introduction

The Achilles tendon (AT) is the largest and strongest tendon in the human body, and it serves as a key structure for elastic energy recoil and force transmission during walking and running (1). However, repeated high cyclic loading, coupled with limited regenerative capacity, predisposes the tendon to load-related degeneration. AT is one of the most commonly injured tendons, with a reported incidence of 2.35 cases of AT injury per 1,000 adults each year in North America (2, 3). The socioeconomic burden associated with AT rupture is substantial, with treatment costs estimated at €6,500 per case in Europe and up to $21,000 in the United States (4). Clinically meaningful adaptation involves both tendon morphology and mechanics, which jointly determine how the tendon distributes stress, stores and returns elastic energy, and tolerates repeated loading (5). Adequate load stimulates collagen synthesis and matrix reorganization, thereby increasing tendon stiffness and resilience (6, 7), whereas insufficient or excessive load can provoke maladaptive changes and increase the risk of tendinopathy or rupture (8). Consequently, determining which exercise interventions and doses most effectively target AT morphology and mechanical properties has become a central objective in sports medicine and rehabilitation.

Exercise-based programs are the primary conservative treatment for tendinopathy and are recommended as first- or second-line interventions (9). A variety of training modalities, such as resistance training, endurance training, plyometric training, or blood flow restriction training, have been shown to produce favorable morphological and mechanical adaptations in ATs (10–19). However, existing evidence is fragmented across modalities (eccentric, heavy–slow resistance, isometric, plyometrics, gait retraining, BFR, etc.) and dosing parameters (intensity, frequency, duration, progression), making it difficult to identify which prescriptions most consistently improve specific tendon outcomes (10, 20). Existing systematic reviews and traditional meta-analyses have relied primarily on methodological consistency across randomized controlled trials (RCTs), including the comparability of intervention and control groups. Furthermore, methodological heterogeneity between RCTs, including differences in participant characteristics, intervention intensity, outcome definitions, and imaging protocols, has complicated data synthesis and limited the clinical applicability of the findings from previous meta-analyses.

Network meta-analysis (NMA) addresses these challenges by combining direct and indirect evidence across multiple interventions, enabling comparative effectiveness estimates and probabilistic rankings even without extensive head-to-head trials (21). Unlike pairwise meta-analysis, NMA can integrate fragmented data from disparate RCTs, thereby enabling comprehensive comparisons even in the absence of head-to-head trials (22–24). Moreover, NMA enables simultaneous comparison and ranking across multiple interventions, although rankings should be interpreted alongside effect sizes and uncertainty.

Therefore, the aim of this systematic review and NMA was to quantify and compare the effects of periodic exercise-based protocols on the AT morphological (e.g., cross-sectional area, length) and mechanical outcomes (e.g., strain, elongation, force, stress, stiffness) among healthy adults. We further sought to identify dose and duration windows that preferentially improve morphology and mechanics, providing pragmatic, evidence-informed starting points for context-specific prevention and early rehabilitation.

## 2 Methods

This review was conducted in accordance with the Cochrane Handbook and the Preferred Reporting Items for Systematic Reviews and Meta-Analyses (PRISMA) guidelines (25, 26). The review protocol is available from PROSPERO (https://www.crd.york.ac.uk/prospero), under the registration number of CRD420251009672.

### 2.1 Study sources and search strategy

PubMed, Web of Science, Scopus, and EMBASE were searched from inception to February 18, 2025. The following search strategy was adapted for each database and applied to the title, abstract, and keyword search fields: (Achilles tendon) AND ((train*) OR (training*) OR (intervention*) OR (exercise*) OR (technique*) OR (protocol*) OR (program*)). No language restrictions were applied at the search stage, however, only English-language full-text articles were eligible for inclusion. Finally, only English full-text articles were ultimately included. No additional filters or search restrictions were applied. Additionally, the reference lists of the included studies and relevant reviews, systematic reviews, and meta-analyses were checked. Any potentially relevant studies identified from reference lists that were not captured by the database search were screened using the same title/abstract process.

### 2.2 Inclusion and exclusion criteria

Following the database searches, all titles and abstracts were imported into an Endnote file (version 21; Thomson Reuters, Carlsbad, CA), and duplicate records were removed using both an automated process and manual verification. The remaining articles were independently assessed for relevance by two reviewers (FBS and QYH). The reviewers independently assessed the full texts of all the articles based on the eligibility criteria. Disagreements between the two reviewers were resolved by consulting a third reviewer (ZXN).

The inclusion criteria were developed based on the Participants, Interventions, Comparators, Outcomes, and Study Design (PICOS) framework. To ensure that the changes in the AT remained within a normal and adaptable range, the population group of interest was healthy adults aged 18–55 years with no history of musculoskeletal injuries or mental health conditions. The relevant interventions included exercise-based training programs for ATs, and none of these studies reported any injuries caused by the interventions. Interventions had to last ≥4 weeks, set as the minimum duration for a periodic exercise intervention [≥3 weeks for stretching interventions (27–29)] with a frequency of at least once per week. Intervention types were classified as follows (with subcategories based on loading characteristics and duration): (1) stretching training, (2) gait retraining (transition the foot-strike pattern from rearfoot strike to forefoot strike in runners), (3) strength training, (4) multimodal training and (5) endurance training. Stretching training was further divided into (1) proprioceptive neuromuscular facilitation (PNF) stretching training, (2) static stretching training, and (3) ballistic stretching training. Multimodal training was further divided into (1) neuromuscular electrical stimulation (NMES) with isometric plantar flexion contraction, (2) low-load blood flow restriction training (LL-BFR), (3) instrument-assisted soft tissue mobilization (IASTM) and (4) specific collagen peptide (SCP) supplementation combined with resistance training. Multimodal interventions were treated as distinct intervention nodes in the network rather than being disaggregated and attributed to a single component. Strength training was further divided into (1) short-term plyometric jump training (PJT), (2) long-term PJT, (3) short-term isometric plantar flexion contraction training, (4) long-term isometric plantar flexion contraction training, (5) short-term eccentric contraction training, and (6) long-term eccentric contraction training. Endurance training was further divided into (1) basic infantry training and (2) habitual running training. We classified interventions lasting more than 12 weeks as long-term, whereas those lasting 12 weeks or less were considered short-term based on the criteria established by Mak et al. (30). The control groups included individuals receiving either placebo administration or maintaining baseline physical activity without any additional training. Control conditions included (i) nontraining/usual-activity controls, (ii) sham/placebo controls, and (iii) within-participant comparators using the contralateral limb or a symmetric non-treated region in unilateral intervention paradigms. To avoid unit-of-analysis errors, within-participant comparators were not combined with parallel-group controls in the primary pairwise meta-analyses and network meta-analyses; such studies were summarized descriptively and explored in sensitivity analyses when sufficient data were available. Studies had to report at least one of the following AT-related outcomes: length (rest length), cross-sectional area (CSA), stiffness, elongation, stress, strain, or force. According to existing studies on the definition of the AT (31), this review adopted the two most commonly used definitions: the AT, defined as the length from the medial gastrocnemius muscle-tendon junction to the insertion into tuber calcaneus; and the free AT, defined as the length from the soleus muscle-tendon junction to the same insertion point. In terms of study design, longitudinal RCTs and longitudinal non-RCT intervention studies were eligible for systematic review. Only RCTs were included in the NMA.

The exclusion criteria were as follows: (a) subjects participated in additional non-exercise interventions (e.g., surgical procedures, pharmacological treatments); (b) interventions did not specifically target AT exercise/training; (c) duplicate data from the same cohort; (d) reviews, conference literature or case studies; or (e) studies published in languages other than English. Eligible studies were required to provide both baseline and postintervention data in numerical or digitizable graphical form, enabling data extraction for analysis.

No publication-date restriction was applied during the search; however, all eligible included studies were published between January 2000 and February 2025.

### 2.3 Study selection

The following data were extracted by two independent reviewers (FBS and QYH): (1) author and publication year; (2) participant characteristics (e.g., sample size, sex ratio, age, height, weight, occupation, or other demographics); (3) intervention characteristics (e.g., type, duration, frequency, and training specifics); and (4) AT outcome measures. The mean change values and standard deviations (SDs) were extracted for both the intervention and control groups from pre- and postintervention measurements. A third reviewer (ZXN) subsequently verified the accuracy of the extracted data.

For the NMA, the standardized mean difference (SMD) was calculated using the mean change value and SD change value. When studies reported baseline and follow-up means and SDs but not change scores, the change SD was computed as follows:.

$$\begin{array}{c} SD_{change}=\sqrt{SD_{\;pre}^{2}+SD_{\;post}^{2}-2R\times SD_{\;pre}\times SD_{\;post}} \end{array}$$

with a correlation coefficient R = 0.5 (32). If standard errors (SEs) rather than SDs were reported, SEs were converted to SDs in accordance with the Cochrane Handbook guidelines (Sect. 6.5.2.2), ensuring that the SE referenced was within-group rather than between-group. When necessary data were missing, the corresponding authors were contact via email up to three times. If no response was received or if the full dataset was unavailable, numerical values were digitized from the figures via the Web Plot Digitizer v4.5 (https://automeris.io) (33). Two reviewers independently performed digitization; discrepancies > 5% were resolved by consulting a third party and taking average values. Studies without extractable numerical or digitizable data were excluded from the NMA. For studies reporting multiple post-intervention time points, all relevant time points were extracted. For quantitative synthesis, the time point closest to the end of the intervention was used in the primary analyses to avoid double-counting participants; additional follow-up time points were summarized descriptively. For studies with multiple intervention subgroups, subgroup means and SDs were combined in accordance with the Cochrane Handbook (Sect. 23.3.4) before pooling with the next subgroup. This approach was used to derive a single, representative effect estimate for each intervention node, thereby reducing heterogeneity arising from within-study variation in intervention intensity, dose, or progression and improving the comparability of loading prescriptions across nodes in the network. The data extraction form and procedures were pilot tested on 10 randomly selected eligible studies. Any ambiguities were resolved, and the template was refined before full extraction.

### 2.4 Risk of bias assessment

Two reviewers (FBS and QYH) independently assessed the risk of bias of each included study. When there was no consensus regarding the risk of bias, a third reviewer (ZXN) was consulted to resolve the disagreement. Because a scale to assess the risk of bias does not exist for evaluating different study designs and biomechanical studies, a validated, modified version of the Downs and Black Quality Index (DBQI) was used to assess the risk of bias across RCTs and non-RCTs (34). The original DBQI comprises 27 items. We omitted five items (Items 9, 13, 17, 18, 19 and 26; Supplementary Table S1) that primarily address attrition/time-related issues and adherence procedures, which are less applicable to short-term laboratory-based exercise trials. Because participant blinding is generally infeasible in exercise-based interventions (35), the blinding-related item in the original DBQI was adapted. Item 15 in the modified DBQI assesses whether a familiarization session was conducted prior to the intervention/testing procedures (Supplementary Table S1). The resulting 22-item tool covers four domains: information reporting (items 1–9), external validity (items 10–11), internal validity (items 12–16), and selection bias (items 17–22). Each item was scored “1” for low risk of bias (clear reporting/adequate method) or “0” for high risk of bias (absent or unclear). The total scores ranged from 0 to 22, with scores of 0–7 indicating high risk of bias, scores of 8–15 indicating a moderate risk of bias, and scores of 16–22 indicating a low risk of bias (Supplementary Table S1).

### 2.5 Statistical analysis

The mean changes and SDs extracted from the included studies were analyzed using Stata 18.0 (Stata Corp LLC, USA) to calculate the SMDs and corresponding 95% confidence intervals (95% CIs). The SMD was expressed as Hedges' g, an adjusted effect size similar to Cohen's d but adjusted for small sample sizes. It was anticipated that there would be heterogeneity between studies; therefore, pooled effect sizes were estimated using a random effects model. Because all outcomes of interest were continuous or ordinal but measured on different scales, the SMD was adopted as the principal effect estimate.

A network geometry was constructed to illustrate and compare all included training interventions. For networks with closed loops, global inconsistency was assessed; a *p* value ≥ 0.05 indicated no evidence of inconsistency and the consistency model was used. If *p* < 0.05, an inconsistency model was considered. Local inconsistency was explored using node-splitting where applicable. For networks without closed loops, inconsistency cannot be evaluated because direct and indirect evidence cannot be contrasted; therefore, a consistency model was fitted.

Pairwise random effects meta-analysis was then conducted to compare any two training protocols. Pairwise analyses were performed only when at least two studies informed a given direct comparison; otherwise, findings were summarized narratively. After assessing comparative effectiveness, surface under the cumulative ranking curves (SUCRAs) were used to calculate the overall rankings for each training protocol in the NMA. The SUCRA ranged from 0% (worst treatment) to 100% (best treatment). It should be emphasized that SUCRA values reflect the probability of relative ranking rather than clinical magnitude or certainty and should not be interpreted as definitive superiority. The SUCRA were interpreted alongside effect estimates vs. nontraining control and their 95% CIs; where the 95% CI did not include 0 and the direction favoured the intervention, the intervention was considered to show a statistically larger improvement than control. For all outcomes, we reported effect estimates (with 95% CIs) and SUCRA rankings; for outcomes with closed loops, we additionally reported inconsistency assessments (global and, where feasible, local). Because heterogeneity existed within intervention nodes, we assessed the sensitivity of the findings by sequentially excluding individual studies and repeating each network meta-analysis.

## 3 Results

### 3.1 Study characteristics

**Figure 1:**
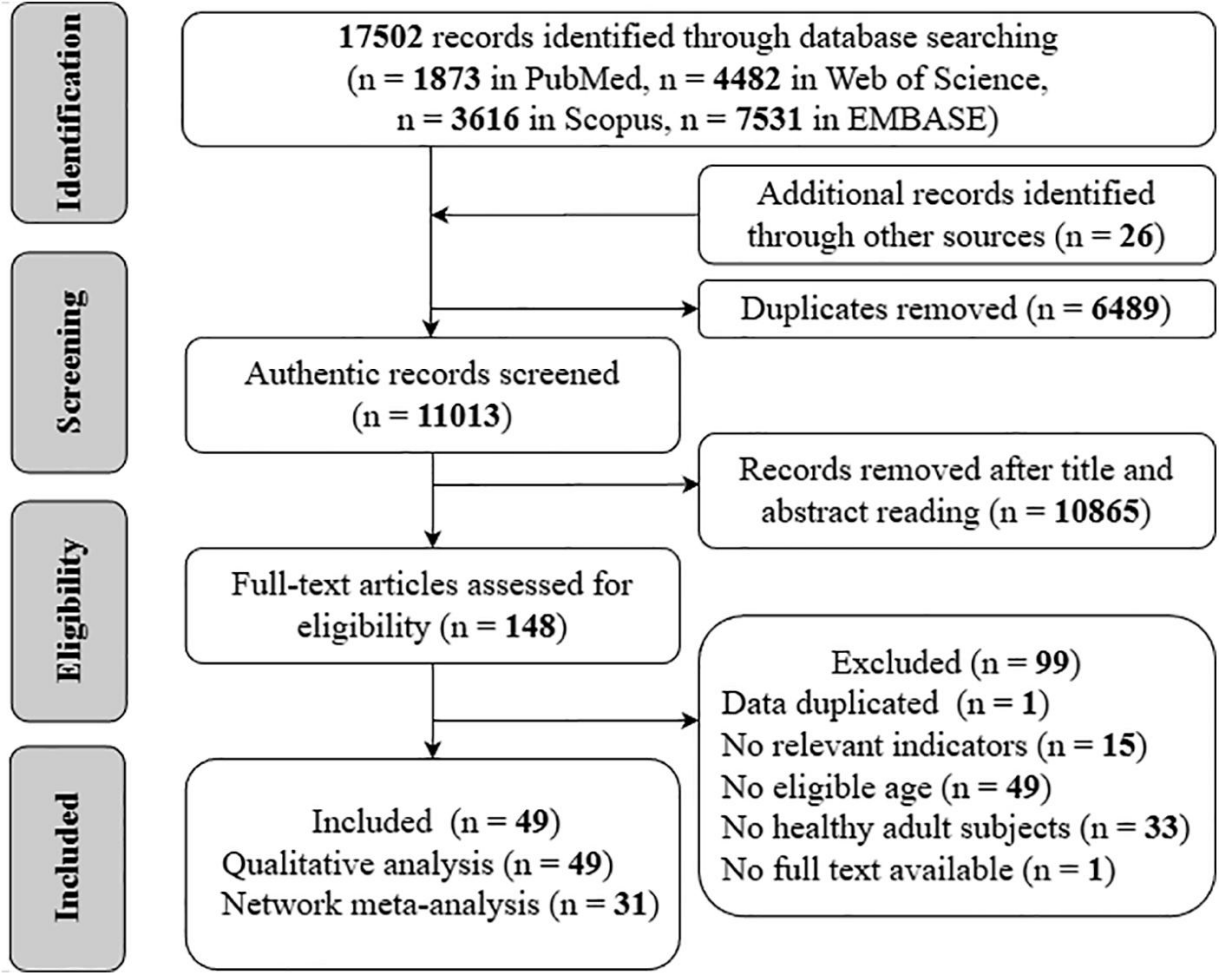
Flowchart of the search process for studies.

A total of 17,502 records were retrieved from PubMed, Web of Science, Scopus, and EMBASE. A manual search of the reference lists of 17 previous systematic reviews yielded 26 additional records. After removing duplicates, 11,013 unique records remained for screening. Title and abstract screening excluded 10,865 records, leaving 148 records for full-text review. Of these, 99 were excluded for failing to meet the inclusion criteria. Therefore, 49 studies (11–13, 17, 36–80) were included in the systematic review. Thirty-one of these studies (63.3%) (11–13, 38–52, 54–62, 64, 68, 71, 73, 75, 77–80) met the criteria for NMA (Figure 1). Pairwise random-effects meta-analyses were conducted when at least two studies informed a direct comparison; otherwise, findings were summarized narratively.

Supplementary Tables S2–S6 summarise the characteristics of the included studies. Publication years ranged from 2003 to 2024. Sample sizes ranged from 8 to 81 participants, intervention duration from 3 to 36 weeks, and training frequency from 1 to 7 sessions per week. Across the 49 studies (*N* = 1,388), interventions were categorised into five training categories comprising 15 subtypes.

The mean risk-of-bias score was 15.4. No study was classified as high risk of bias (0–7). Twenty-six studies were rated as moderate risk of bias (8–15) and 23 as low risk of bias (16–22). The most frequently rated high/unclear items were: adverse event reporting, examiner blinding, participant familiarity with training, randomisation in testing, adjustment for confounders, and reporting of sample-size calculations (Supplementary Table S7).

**Figure 2:**
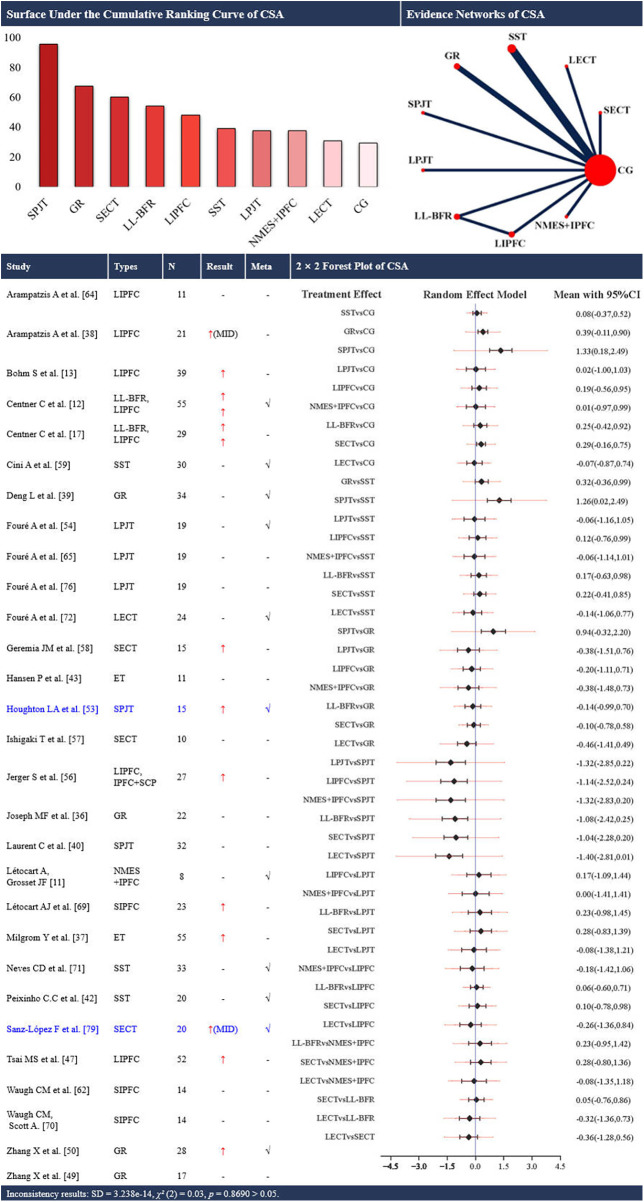
Effects of different types of training on the cross-sectional area of the achilles tendon. CSA, cross-sectional area; CG, control group; SST, static stretching training; SPJT, short-term plyometric jump training; LPJT, long-term plyometric jump training; SIPFC, short-term isometric plantar flexion contraction; LIPFC, long-term isometric plantar flexion contraction; SECT, short-term eccentric contraction training; LECT, long-term eccentric contraction training; GR, gait retraining; NMES + IPFC, neuromuscular electrical stimulation with isometric plantar flexion contraction; LL-BFR, low-load blood ﬂow restriction training; IPFC+SCP, specific collagen peptide supplementation combined with resistance training; ET, endurance training; MID, middle of the tendon; CI, confidence interval; -, no statistically significant change/exclusion from the network meta-analysis; ↑, significantly increased; √, included in the network meta-analysis. In the evidence networks of CSA, the size of the nodes relates to the number of participants in that intervention type, and the thickness of lines between interventions relates to the number of studies for that comparison. Blue font indicates interventions that rank highly but are supported by a single study (single-study nodes).

**Figure 3:**
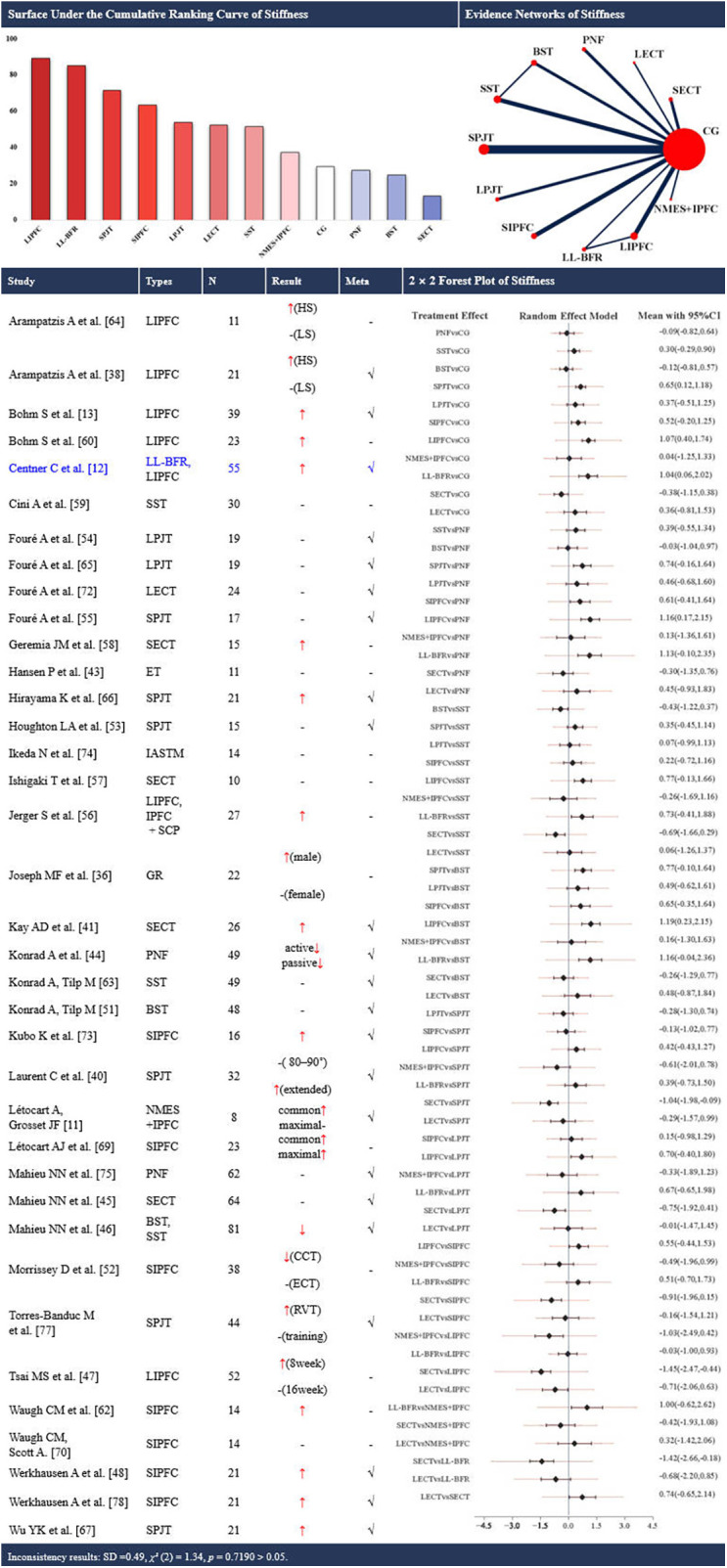
Effects of different types of training on the stiffness of the achilles tendon. CG, control group; PNF, proprioceptive neuromuscular facilitation stretching; SST, static stretching training; BST, ballistic stretching training; SPJT, short-term plyometric jump training; LPJT, long-term plyometric jump training; SIPFC, short-term isometric plantar flexion contraction; LIPFC, long-term isometric plantar flexion contraction; SECT, short-term eccentric contraction training; LECT, long-term eccentric contraction training; GR, gait retraining; NMES + IPFC, neuromuscular electrical stimulation with isometric plantar flexion contraction; LL-BFR, low-load blood ﬂow restriction training; IASTM, instrument-assisted soft tissue mobilization; IPFC+SCP, specific collagen peptide supplementation combined with resistance training; ET, short-term eccentric contraction training; HS, high-strain group; LS, low-strain group; common, elongation at common force; maximal, elongation at maximal force; RVT, reduced-volume training; training; training, nonreduced-volume training; CCT, concentric contraction training; ECT, eccentric contraction training; CI, confidence interval; -, no statistically significant change/exclusion from the network meta-analysis; ↑, significantly increased; ↓, significantly decreased; √, included in the network meta-analysis. In the evidence networks of length, the size of the nodes relates to the number of participants in that intervention type, and the thickness of lines between interventions relates to the number of studies for that comparison. Blue font indicates interventions that rank highly but are supported by a single study (single-study nodes).

**Figure 4:**
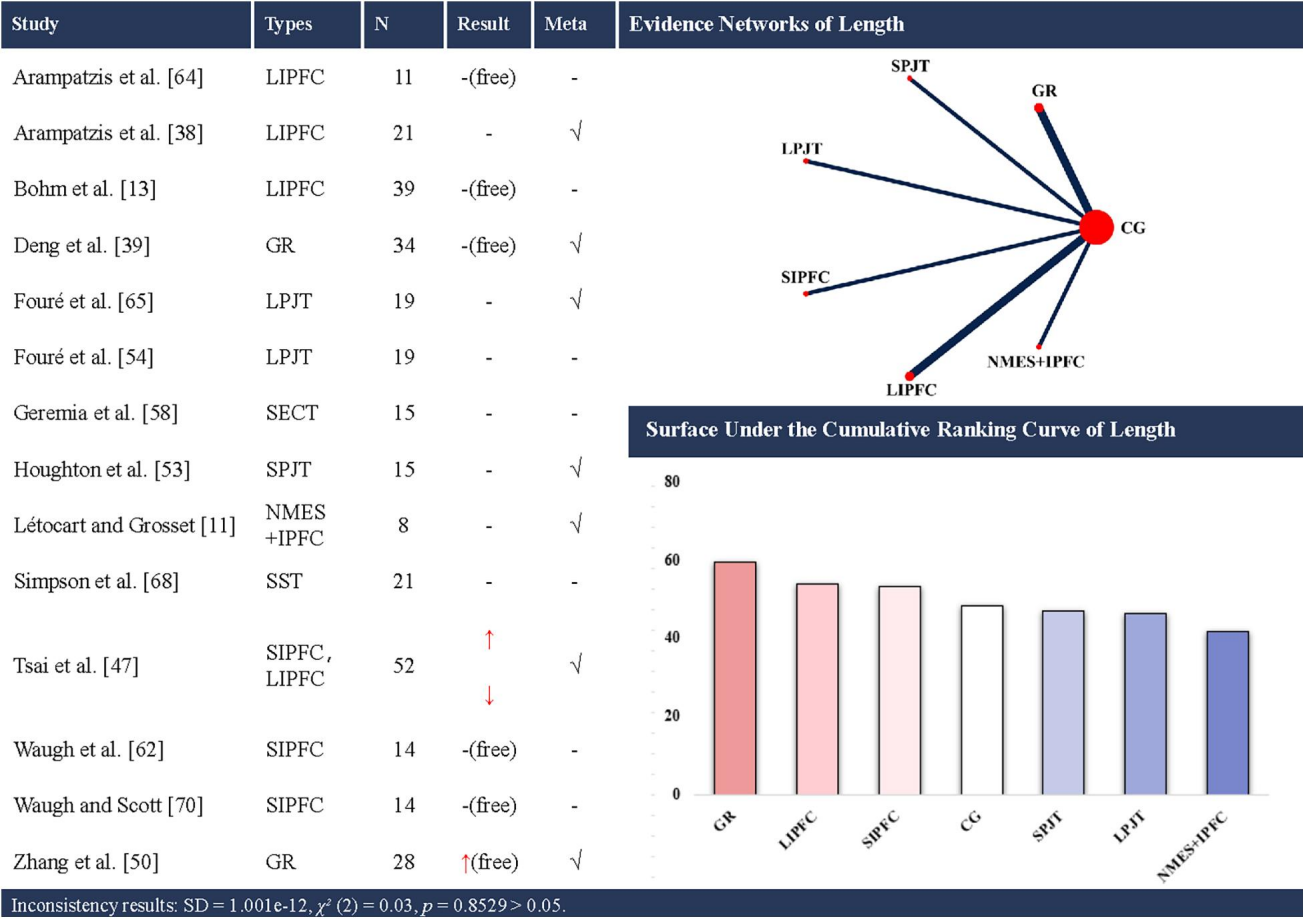
Effects of different types of training on the length of the achilles tendon. CG, control group; SST, static stretching training; SPJT, short-term plyometric jump training; LPJT, long-term plyometric jump training; SIPFC, short-term isometric plantar flexion contraction; LIPFC, long-term isometric plantar flexion contraction; SECT, short-term eccentric contraction training; GR, gait retraining; NMES + IPFC, neuromuscular electrical stimulation with isometric plantar flexion contraction; free, free Achilles tendon; -, no statistically significant change/exclusion from the network meta-analysis; ↑, significantly increased; ↓, significantly decreased; √, included in the network meta-analysis. In evidence networks of length, the size of the nodes relates to the number of participants in that intervention type, and the thickness of lines between interventions relates to the number of studies for that comparison.

**Figure 5:**
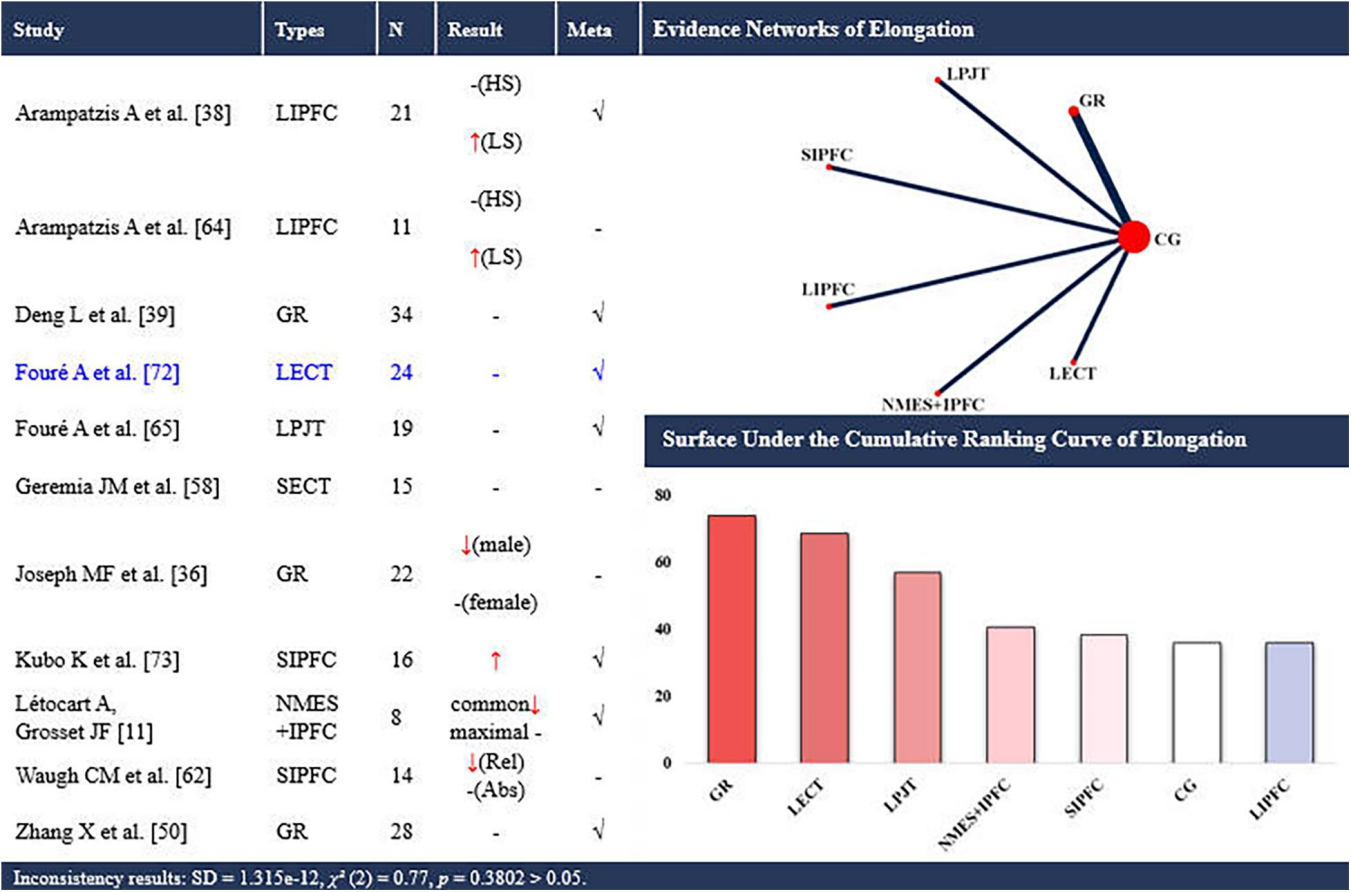
Effects of different types of training on the elongation of the achilles tendon. CG, control group; LPJT, long-term plyometric jump training; SIPFC, short-term isometric plantar flexion contraction; LIPFC, long-term isometric plantar flexion contraction; SECT, short-term eccentric contraction training; LECT, long-term eccentric contraction training; GR, gait retraining; NMES + IPFC, neuromuscular electrical stimulation with isometric plantar flexion contraction; HS, high-strain group; LS, low-strain group; common, elongation at common force; maximal, elongation at maximal force; Rel, relative elongation; Abs, absolute elongation; -, no statistically significant change/exclusion from the network meta-analysis; ↑, significantly increased; ↓, significantly decreased; √, included in the network meta-analysis. In evidence networks of length, the size of the nodes relates to the number of participants in that intervention type, and the thickness of lines between interventions relates to the number of studies for that comparison. Blue font indicates interventions that rank highly but are supported by a single study (single-study nodes).

**Figure 6:**
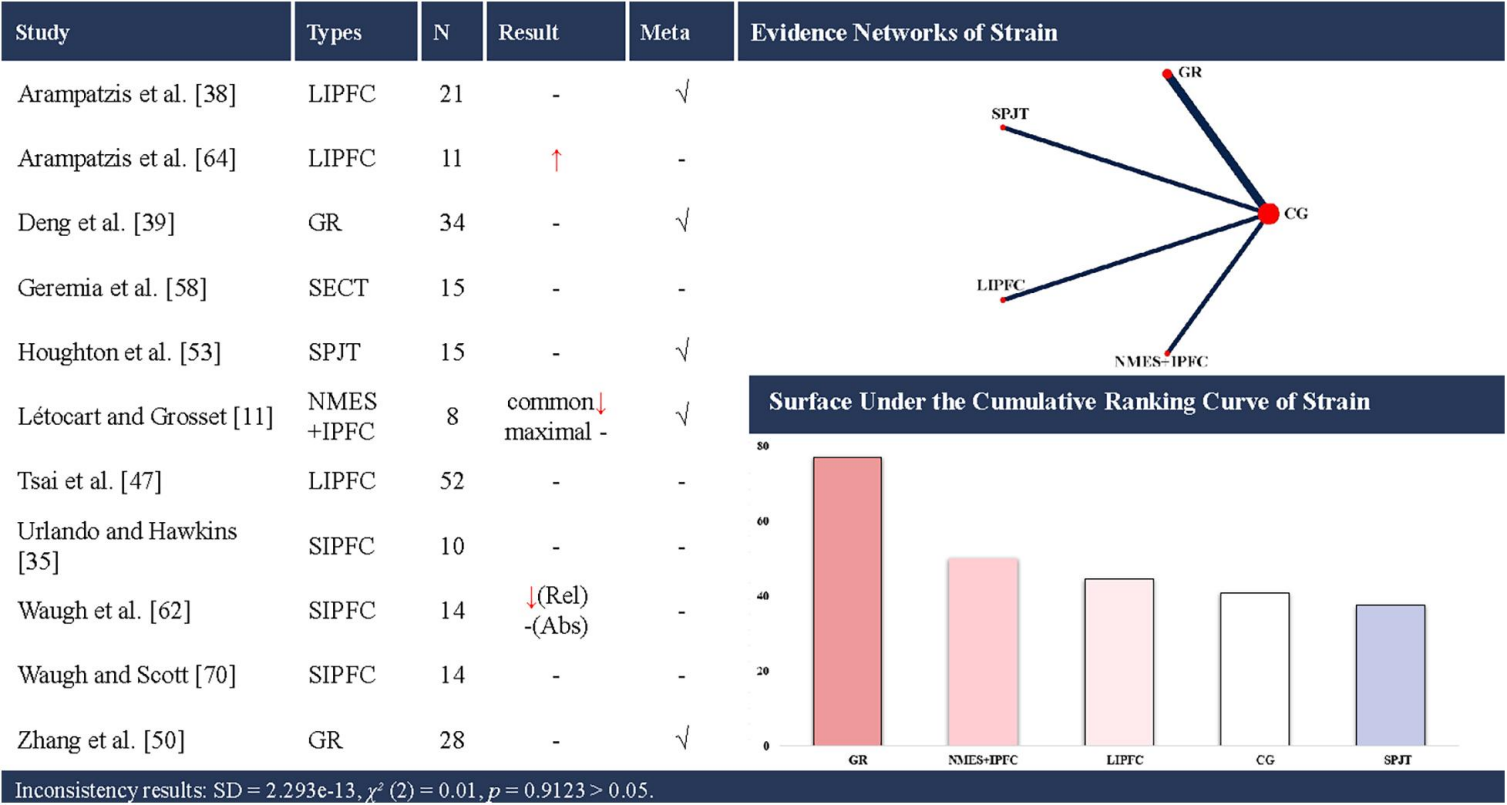
Effects of different types of training on the strain of the achilles tendon. CG, control group; SPJT, short-term plyometric jump training; SIPFC, short-term isometric plantar flexion contraction; LIPFC, long-term isometric plantar flexion contraction; SECT, short-term eccentric contraction training; GR, gait retraining; NMES + IPFC, neuromuscular electrical stimulation with isometric plantar flexion contraction; common, elongation at common force; maximal, elongation at maximal force; Rel, strain at relative elongation; Abs, strain at absolute elongation; -, no statistically significant change/exclusion from the network meta-analysis; ↑, significantly increased; ↓, significantly decreased; √, included in the network meta-analysis. In evidence networks of length, the size of the nodes relates to the number of participants in that intervention type, and the thickness of lines between interventions relates to the number of studies for that comparison.

**Figure 7:**
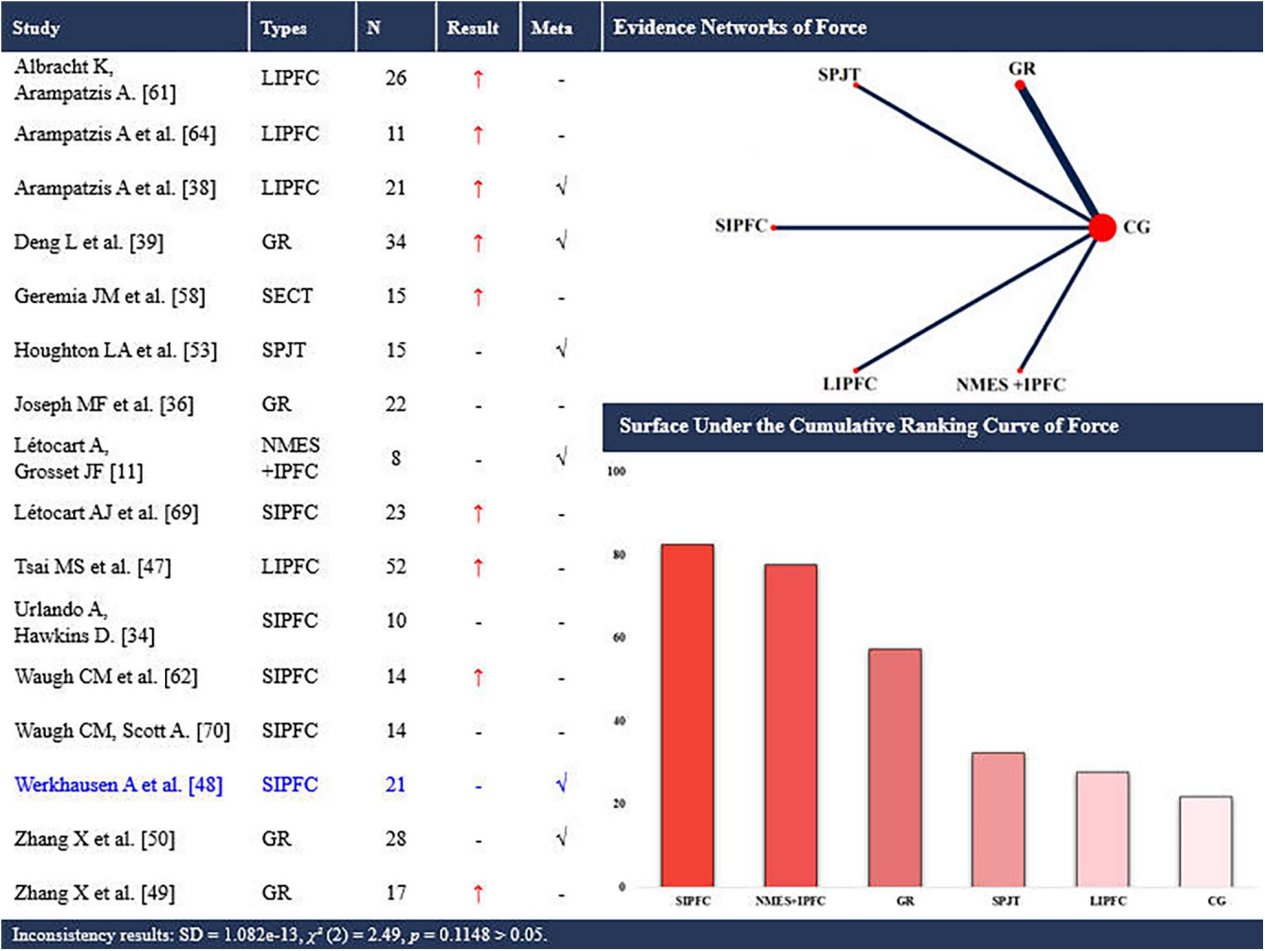
Effects of different types of training on the force on the achilles tendon. CG, control group; SPJT, short-term plyometric jump training; SIPFC, short-term isometric plantar flexion contraction; LIPFC, long-term isometric plantar flexion contraction; SECT, short-term eccentric contraction training; GR, gait retraining; NMES + IPFC, neuromuscular electrical stimulation with isometric plantar flexion contraction; -, no statistically significant change/exclusion from the network meta-analysis; ↑, significantly increased; √, included in the network meta-analysis. In evidence networks of length, the size of the nodes relates to the number of participants in that intervention type, and the thickness of lines between interventions relates to the number of studies for that comparison. Blue font indicates interventions that rank highly but are supported by a single study (single-study nodes).

**Figure 8:**
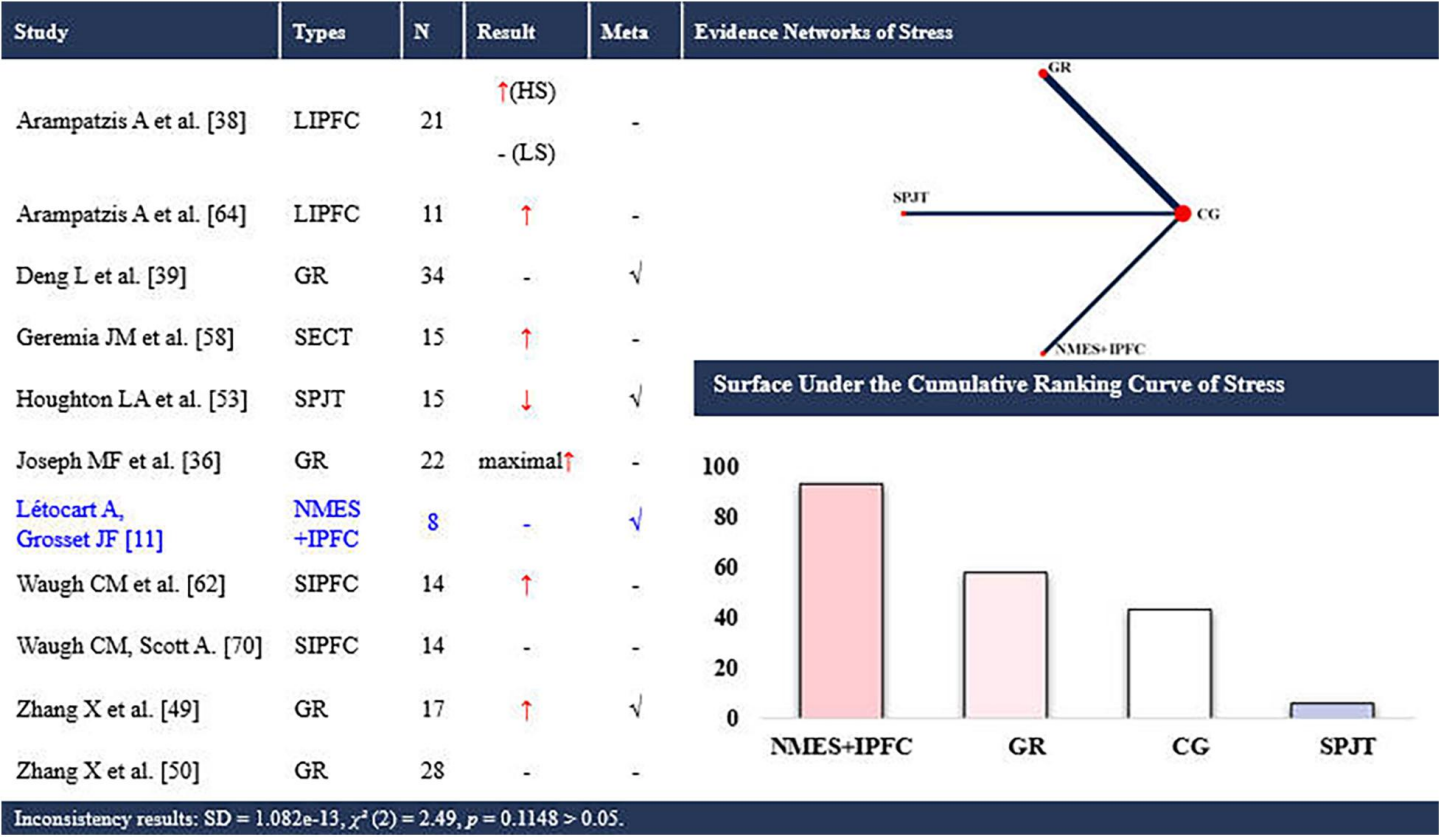
Effects of different types of training on the stress of the achilles tendon. CG, control group; SPJT, short-term plyometric jump training; SIPFC, short-term isometric plantar flexion contraction; LIPFC, long-term isometric plantar flexion contraction; SECT, short-term eccentric contraction training; GR, gait retraining; NMES + IPFC, neuromuscular electrical stimulation with isometric plantar flexion contraction; HS, high-strain group; LS, low-strain group; maximal elongation at maximal force; -, no statistically significant change/exclusion from the network meta-analysis; ↑, significantly increased; ↓, significantly decreased; √, included in the network meta-analysis. In evidence networks of length, the size of the nodes relates to the number of participants in that intervention type, and the thickness of lines between interventions relates to the number of studies for that comparison. Blue font indicates interventions that rank highly but are supported by a single study (single-study nodes).

Network structure note. Closed loops were present only for cross-sectional area (CSA) (Figure 2) and stiffness (Figure 3). For all other outcomes (Figures 4–8), networks contained no closed loops. Therefore, inconsistency could not be empirically assessed and results were interpreted cautiously. In addition, for each outcome, sequential leave-one-out analyses with repeated NMAs showed no indications of outliers or overly influential studies.

### 3.2 CSA of the AT

Twenty-nine studies (11–13, 17, 37–41, 43, 44, 48, 50, 51, 54, 55, 57–60, 63, 65, 66, 70–73, 77, 80) using 12 different interventions investigated the effects of training on the tendon CSA. Eleven of these studies (11, 12, 40, 43, 51, 55, 59, 60, 72, 73, 80) were eligible for NMA, involving 9 types of intervention (Figure 2). A total of 716 participants (600 males and 116 females) were included.

In the NMA, the evidence plot for the CSA of the AT revealed a closed loop (Figure 2). No statistical evidence of inconsistency was observed [SD = 3.238e-14, *χ*^2^(2) = 0.03, *p* = 0.87]. Node-splitting analyses indicated no local inconsistency (all *p* > 0.05). Therefore, results from the consistency model are presented. Short-term PJT ranked highest (SUCRA: 95.6%), whereas long-term eccentric training ranked lowest (SUCRA: 30.9%). Direct evidence from one study suggested greater CSA with short-term PJT vs. control (SMD [95% CI]: 1.33 [0.18 to 2.49]; Figure 2).

### 3.3 AT length

Fourteen studies (11, 13, 39, 40, 48, 50, 54, 55, 59, 63, 65, 66, 69, 71) examined the effects of training on AT length, encompassing 8 distinct interventions. Seven of these studies (11, 39, 40, 48, 50, 54, 55) were eligible for NMA, involving 6 interventions (Figure 4). A total of 310 participants (268 males and 42 females), were included. The consistency model suggested that gait retraining (SUCRA: 59.6%), long-term isometric plantar flexion contraction (SUCRA: 53.8%) and short-term isometric plantar flexion contraction (SUCRA: 53.2%) ranked highest for increasing AT length (up to +24.19 mm), whereas NMES with isometric plantar flexion contraction (SUCRA: 41.7%), long-term PJT (SUCRA: 46.3%) and short-term PJT (SUCRA: 47.1%) ranked lowest.

### 3.4 AT elongation

Eleven studies (11, 37, 39, 40, 51, 59, 63, 65, 66, 73, 74) examined the effects of training on AT elongation, encompassing 7 distinct interventions. Seven of these studies (11, 39, 40, 51, 66, 73, 74) were eligible for NMA, involving 6 interventions (Figure 5). A total of 212 participants (162 males and 50 females) were included. The consistency model suggested that gait retraining (SUCRA: 74.0%) and long-term eccentric contraction training (SUCRA: 68.5%) ranked highest for increasing elongation (up to +4.7 mm), whereas long-term isometric plantar flexion contraction ranked lowest (SUCRA: 35.9%).

### 3.5 AT strain

Eleven studies (11, 36, 39, 40, 48, 51, 54, 59, 63, 65, 71) examined the effects of periodic training on AT strain, encompassing 7 distinct interventions. Five of these studies (11, 39, 40, 51, 54) were eligible for the NMA of the AT strain, involving 4 interventions (Figure 6). A total of 222 participants (180 males and 42 females) were included. The consistency model suggested that gait retraining (SUCRA: 77.1%) ranked highest for increasing AT strain (up to +3.8%), whereas short-term PJT ranked lowest (SUCRA: 37.6%).

### 3.6 AT force

Sixteen studies (11, 36, 37, 39, 40, 48–51, 54, 59, 62, 63, 65, 70, 71) examined the effects of periodic training on AT force, encompassing 6 distinct interventions. Six of these studies (11, 39, 40, 49, 51, 54) were eligible for the NMA of AT force, involving 5 interventions (Figure 7). A total of 331 participants (264 males and 67 females) were included. The consistency model suggested that short-term isometric plantar flexion contraction (SUCRA: 82.5%) and NMES with isometric plantar flexion contraction (SUCRA: 77.8%) ranked highest for increasing AT force (up to +763.17 N), whereas long-term isometric plantar flexion contraction ranked lowest (SUCRA: 27.7%).

### 3.7 AT stress

Eleven studies (11, 37, 39, 40, 50, 51, 54, 59, 63, 65, 71) examined the effects of periodic training on AT stress, encompassing 7 distinct interventions. Four of these studies (11, 40, 50, 54) were eligible for NMA, involving 3 interventions (Figure 8). A total of 199 participants (142 males and 57 females) were included. The consistency model suggested that NMES with isometric plantar flexion contraction ranked highest for increasing AT stress (SUCRA: 93%; up to +17.4 MPa), whereas short-term PJT ranked lowest (SUCRA: 6%).

### 3.8 AT stiffness

Thirty-seven studies (11–13, 37, 39, 41, 42, 44–49, 52–61, 63–68, 70, 71, 73–76, 78, 79) examined the effects of training on AT stiffness, encompassing 15 distinct interventions. Twenty-three of these studies (11–13, 39, 41, 42, 45–47, 49, 52, 54–56, 64, 66–68, 73, 74, 76, 78, 79) were eligible for NMA, involving 11 interventions (Figure 3). A total of 1076 participants (742 males and 334 females) were included.

The NMA plot revealed two closed loops (Figure 3). No statistical evidence of inconsistency was observed [SD = 0.49, *χ*^2^ (2) = 1.34, and *p* = 0.72]. Node-splitting suggested no statistically significant local inconsistency (all *p* > 0.05). Long-term isometric plantar flexion contraction training (SUCRA: 89.3%) and LL-BFR (SUCRA: 85.2%) ranked highest in probabilistic rankings for improving AT stiffness, whereas short-term eccentric training (SUCRA: 13.2%), ballistic stretching (SUCRA: 24.8%), and PNF (SUCRA: 27.5%) ranked lowest. Pairwise meta-analysis further revealed that short-term PJT (SMD [95% CI]: 0.65 [0.12, 1.18], 5 studies), long-term isometric plantar flexion contraction (SMD [95% CI]: 1.07 [0.40, 1.74], 3 studies), and LL-BFR (SMD [95% CI]: 1.04 [0.06, 2.02], 1 study) resulted in increased AT stiffness (up to +203.33 N/mm) compared with the control group (Figure 3).

## 4 Discussion

The aim of this systematic review and NMA was to investigate the effectiveness of specific exercise-based interventions on AT adaptation among healthy adults. A total of 49 studies (including 30 network meta-analyses) were included, comprising 1,388 participants and 15 subcategories of exercise-based interventions. To improve comparability across studies, we aggregated clinically recognizable and methodologically similar interventions to characterise plausible dose–duration ranges rather than to prescribe a single dose. When studies reported multiple doses or subgroups, we combined subgroup means and SDs following Cochrane guidance. All pooled evidence suggested that targeted mechanical loading, particularly isometric plantar flexion contraction training, gait retraining, and PJT, tended to rank favorably for improving AT morphology (CSA, length) and mechanics (elongation, strain, force, stress, stiffness) in healthy adults (SUCRA > 70%). However, rankings for AT force and stress were informed by sparse evidence and should therefore be interpreted cautiously. In the included trials, these interventions were commonly implemented using loading or exposure parameters such as 80%–90% MVC for isometrics, 200–400 contacts per week for PJT, and 5–48 min per session for gait retraining. Multimodal training, particularly NMES combined with isometric plantar flexion contractions and LL-BFR, also yielded comparatively large gains in force, stress, and stiffness, whereas stretching-focused protocols (static, PNF, ballistic) and short-term eccentric regimens generally ranked low across outcomes (SUCRA < 30%). These rankings reflect probabilities of relative superiority rather than effect magnitude and should be interpreted in light of between-study heterogeneity in loading patterns, program duration, outcome definitions, and baseline activity levels. Accordingly, the apparent advantage of progressive, higher-dose prescriptions requires confirmation in adequately powered, head-to-head randomized trials with harmonized training definitions and standardized outcome measures. The present findings are derived from studies in healthy, asymptomatic adults aged 18–55 and therefore primarily apply to this population. While the results may inform prevention-oriented conditioning, direct extrapolation to clinical tendinopathy or rehabilitation settings should be undertaken with caution.

### 4.1 AT CSA and length

Across the 27 studies included in this review, 12 distinct training modalities were evaluated for their effects on AT morphology. Approximately 44.4% of these studies reported a significant increase in the CSA of the AT, with interventions such as short-term PJT, gait retraining, and short-term eccentric contraction training showed comparatively larger CSA responses when delivered at 55%–90% MVC/55%–85% 1RM, 2–4 sets of 3–5 repetitions per week for 6–16 weeks with progression, among which 71.4% of isometric plantar flexion contraction interventions achieved effective outcomes at 80%–90% MVC, 3–4 sessions per week, for 8–14 weeks. Protocols that pair sufficiently higher strain magnitude and rate with greater weekly time-under-tension, sufficient program duration, and progression, tend to yield larger CSA gains than interventions with lower strain magnitude, lower strain rate, or inadequate exposure. Plyometrics supply high peak and rate stimuli, gait retraining raises cumulative weekly exposure during habitual running, and short-term eccentric contraction training at lengthened positions concentrates load in the mid-portion (39). In contrast, changes in tendon length were less commonly observed. Changes in tendon length may require sustained high-tension loading and longer intervention durations, because longitudinal remodeling depends on interfascicular sliding and, potentially, insertional adaptations. Only two studies, namely, one involving short-term isometric plantar flexion contraction training at 90% MVC, with 3 s loading, 3 s relaxing per contraction, 4 sets of 5 repetitions, for 12 weeks and one gait retraining trial with forefoot strike pattern practiced for 3 sessions per week, 5–48 min per session, for 12 weeks, reported increased AT length (48, 51). These morphological adaptations are thought to occur only when mechanical loading surpasses a threshold strain capable of stimulating collagen synthesis and remodelling (65, 81, 82). Whether a protocol surpasses the putative strain threshold likely depends on the combination of intensity, contraction duration, weekly time-under-tension, program length, and progression. Protocols falling short on one or more dimensions may fail to trigger measurable morphological change. However, high-intensity loads applied over short durations may also lead to transient fluid shifts or localized oedema, potentially inflating CSA measurements (83). The extent of this effect is influenced by the imaging modality and study design.

The NMA ranked short-term PJT and gait retraining as the top interventions for increasing CSA and tendon length, respectively. Notably, these rankings were based on a limited number of trials and heterogeneous measurement approaches, and should be interpreted as relative probabilities rather than definitive superiority. The top ranking of short-term PJT was supported by a single study (180–300 contacts/week, 8 weeks), thereby limiting the generalizability of the results, whereas the SUCRA value for gait retraining was 59.6%, indicating only a moderate probability of superiority. Furthermore, in pairwise comparisons, only short-term PJT vs. the control had a significant effect on CSA (mean = 1.33, 95% CI: 0.18–2.49). Most other comparisons had 95% confidence intervals that crossed zero, likely reflecting small sample sizes in 91% of the studies (< 50 participants), which increased standard errors and hindered statistical power. While the current data suggest a potential benefit of short-term PJT and gait retraining, larger, high-quality RCTs are needed to validate these findings.

This review revealed that the magnitude and regional pattern of AT adaptation are strongly modulated by measurement methods, sampling location, and participants' baseline physical activity levels. Although tendon CSA and length are primarily assessed with ultrasound or MRI, substantial methodological heterogeneity exists across studies regarding body position and the anatomical site of acquisition. Ultrasound measurements can be affected by probe pressure and resolution (84–87), whereas MRI, which offers higher contrast, may suffer from the “magic angle effect”, particularly when the tendon aligns at ≈ 55° to the magnetic field (88–90). Additionally, resting tension and joint positioning impact CSA and length outcomes. The proximal tendon exhibits more lateral deformation under stretch, making location standardization critical (91). Differences in participants' athletic backgrounds also play a role. For example, endurance runners typically show greater region-specific AT hypertrophy than nonrunners do (92). Collectively, these factors underscore the need for standardized measurement protocols and detailed reporting of training history.

### 4.2 AT elongation and strain

Elongation and strain reflect tendon deformation under loading and are commonly interpreted as mechanical indicators related to energy storage, load transmission efficiency, and potential overload risk. However, the “desirable” direction of change is context-dependent. Greater compliance (higher elongation/strain for a given load) may facilitate energy storage and return, whereas reduced strain at a given force can indicate increased stiffness and potentially improved load tolerance. Accordingly, interpretation requires alignment with the training goal and with concurrent changes in force capacity and stiffness.

Across studies, isometric plantar flexion contraction training was the most frequently studied modality for AT elongation, accounting for 36.4% of the included studies. Most studies applying low-strain, cyclic loading reported significant increases in tendon elongation. Cyclic low-strain loading tends to increase apparent elongation because repeated sub-threshold strain cycles promote viscoelastic conditioning and interfascicular sliding, thereby lowering resistance to deformation under a given load. Effects were most apparent when strain amplitude was around 55%–80% MVC, with a time under tension of 15–21 s, weekly exposure of approximately 4 cycles per week, and delivered for 12–14 weeks. These changes are believed to result from microstructural remodelling of collagen fibres under mechanical tension. Compared with higher-intensity protocols, low-strain cyclic work emphasizes conditioning of the matrix and gliding between fascicles, which manifests as greater elongation at an appropriate load. However, one short-term isometric plantar flexion contraction training study and one gait retraining study reported decreases in elongation, potentially due to strain rate sensitivity or sex-specific responses (37, 63). While 54.5% of the studies assessing strain involved isometric plantar flexion contraction training, most did not report significant changes. Strain estimates were heterogeneous, which likely reflects modest between-group changes under sub-threshold dosing as well as methodological variation in defining resting length, probe fixation/tracking, and task selection. Notably, lower strain of the AT often indicates a reduced capacity for energy storage; however, when lower stress is accompanied by an increased CSA, this may instead favor greater energy storage within the tendon. Therefore, when interpreting whether an increase in AT strain is beneficial for tendon energy storage, it is necessary to consider the corresponding changes in CSA.

Although none of the gait retraining trials showed statistically significant improvements in elongation or strain individually, the NMA ranked it highest for both outcomes. This apparent discrepancy stems from the way ranking probabilities are generated. If several small studies consistently favour gait retraining by a modest margin, even when each estimate is imprecise, the cumulative ranking algorithm will still assign a high probability that gait retraining is “best”. A SUCRA value of 75%–80% does not confirm clinical superiority but suggests a greater likelihood of benefit relative to other modalities. Therefore, this finding needs to be interpreted with caution until larger, adequately powered head‒to‒head trials can confirm whether the small numerical benefits attributed to gait retraining translate into statistically and practically significant tendon adaptations.

### 4.3 AT force and stress

Among the studies included in this review, all 4 investigations of long-term isometric plantar flexion contraction training reported significant increases in AT force, notably when protocols met intensity targets (55%–90% MVC/55% 1RM) and exposure targets (12–16 weeks with progression) (39, 48, 62, 65); of the 5 studies on short-term isometric plantar flexion contraction training, 2 reported significant increases when intensity and exposure thresholds were reached (90% MVC/55% 1RM), with a hold duration of 3 s, for 12 weeks (63, 70); similarly, 2 of the 4 gait retraining trials also demonstrated significant increases in AT force when the protocol was practiced for 5–48 min per session, 3 sessions per week, for 12 weeks (40, 50). Across modalities, the shared pathway is not a particular contraction mode but, within each task context, the delivery of sufficient strain or tension magnitude, adequate weekly exposure (time under tension), and a sustained, progressive program duration. It should be noted that AT stress does not represent force generated by the tendon itself, since the AT does not intrinsically generate force; rather, it reflects the efficiency with triceps surae muscle force is transmitted through the tendon. That is, a higher AT force after training indicates an improvement in the efficiency of force transmission. Isometric plantar flexion contraction training achieves such exposure through high-tension static holds, whereas gait retraining do so through repeated stretch-shortening cycles that impose higher strain rates and peaks. In contrast, AT stress outcomes were derived from 11 studies employing seven different intervention types, resulting in a more diffuse evidence base. Unlike force, AT stress is therefore shaped by both the method used to estimate force and the approach used to measure CSA. Across studies, substantial heterogeneity exists in force estimatio, in CSA acquisition, and in the test task used for evaluation, which amplify differneces between-study dispersion in reported AT stress outcomes. Although most of the studies reported significantly increased stress, one study involving short-term PJT reported a significant reduction in stress (54). This pattern is compatible with relatively faster CSA increases without concurrent force gains under the PJT. Similarly, in the study by Houghton et al. (54), the CSA increased significantly (+ 12.8%) without a corresponding rise in force, leading to reduced stress. Mechanistically, an increase in AT stress primarily depends on the proportional changes in tendon force and CSA, and a significant increase in tendon stress occurs only when the increase in tendon force outweighs that of CSA, which also reflects an increase in tendon loading. Conversely, if CSA expands more rapidly or measurements are affected by acute fluid shifts, stress may remain stable or decline.

Importantly, only one study on short-term isometric plantar flexion contraction training was included (49), and it reported no significant change in force before and after the intervention; however, this modality ranked highest in the probability ranking. This phenomenon may be due to the requirement for ranking stability in NMA; compared with the other interventions, the short-term isometric plantar flexion contraction training group presented a smaller standard deviation of the change scores for force, indicating lower variability and relatively greater stability. Similarly, NMES with isometric plantar flexion contraction ranked highest for stress, despite being supported by a single study that reported nonsignificant results (11). This ranking may be influenced by a large mean change effect and lower baseline physical activity levels in the participants. These findings highlight how statistical rankings can sometimes diverge from direct evidence, thus highlighting the need for cautious interpretation.

Additionally, heterogeneity in measurement techniques complicates comparisons. Across studies, AT force was estimated using diverse approaches, including (i) MVC-based dynamometry (plantar-flexion torque divided by an estimated subject-specific moment arm), (ii) inverse-dynamics estimates during locomotion (ankle moment divided by an estimated instantaneous moment arm), (iii) custom ergometer/strain-gauge setups, and, in rare cases, direct force-transducer recordings used as a proxy for triceps surae force. These approaches rely on differing upstream assumptions (e.g., moment-arm estimation, co-contraction handling, normalization, and CSA measurement), which may introduce systematic between-study variability and should be considered when interpreting pooled outcomes. While reported peak tendon forces were generally within physiologically plausible ranges with no clear outliers, derived stress estimates are particularly sensitive to methodology. The four studies reporting stress used ultrasound-derived CSA measured at different anatomical sites (e.g., 2, 4, or 10 cm proximal to the calcaneal insertion), and because AT CSA varies along its length, site differences can materially affect stress calculations.

### 4.4 AT stiffness

All 7 long-term isometric plantar flexion contraction training studies (12, 13, 39, 48, 57, 61, 65) and the single LL-BFR training study (12) consistently reported significant increases in AT stiffness particularly when protocols met dose anchors of intensity (90%–100% MVC/20%–85% 1RM/80% maximum force), with a hold duration of 1–3 s per contraction, 3–10 sets of 4–20 repetitions per session, 2–4 sessions per week, for 4–16weeks. Similarly, most investigations of short-term isometric plantar flexion contraction training also revealed significantly increased stiffness with significant effects observed when intensity and exposure thresholds were reached (80%–90% MVC/80% maximum force), with a hold duration of 1–3 s, for 10–12 weeks. Similarly, a review by Bohm et al. (20) indicated that increased stiffness is primarily mediated by changes in the tendon modulus, collagen density, and matrix protein proliferation. Isometric plantar flexion contraction training disrupts homeostasis via repetitive mechanical loading, activating fibroblasts and promoting matrix protein synthesis (65, 81). LL-BFR, with cuffs set at 50% arterial occlusion pressure (AOP) and loading at 20%–35% 1RM, using a 3–4 sets of 15–30 repetitions scheme (e.g., 30-15-15-15), 2–3 sessions per week for 14 weeks, enhances this effect by creating a hypoxic environment that accelerates fibroblast proliferation and collagen deposition (93–97). In contrast, stretching exercises (including PNF, static stretching, and ballistic stretching), as well as some PJT and eccentric contraction training protocols, did not significantly change or even reduce stiffness. The stiffness-reducing effect of stretching may be explained by the more complete straightening of collagen crimp patterns and the transient redistribution of water and proteoglycans within the tendon matrix (98–100). Furthermore, the lack of significant stiffness changes observed in several PJT and eccentric contraction training studies may be attributed to factors such as baseline imbalances between the intervention and control groups (66) or increased tendon blood volume induced by exercise (58). However, in those PJT interventions where stiffness increased, 75% of the training doses fell within the range of 200–400 contacts per week for 8–12 weeks. Across modalities, larger stiffness gains are most plausibly a dose-driven phenomenon: protocols that achieve higher tension magnitudes, accumulate more weekly time-under-tension, and sustain a progressive, multi-week schedule tend to up-regulate collagen synthesis and increase tendon modulus, whereas protocols emphasizing low-tension or predominantly viscoelastic conditioning are more likely to maintain or reduce stiffness. However, stiffness should not be interpreted as universally “higher is better.” Increased stiffness may reduce operating strain at a given load, yet overly stiff behavior could alter joint loading patterns and force transmission. Therefore, target stiffness should be individualized by sport demands, training phase, and symptom status.

The SUCRA rankings identified long-term isometric plantar flexion contraction training, LL-BFR, and short-term isometric plantar flexion contraction training as the top three interventions for enhancing tendon stiffness. The control and stretching-dominant conditions (PNF, gait retraining, short-term eccentric contraction training, and control groups) ranked lowest (< 40%). These rankings were consistent with the cumulative mechanical stimulus imposed by each modality, with longer training durations resulting in a greater adaptive load. The pairwise comparison results revealed all three intervention groups presented significant increases for the control group. Notably, only one study on LL-BFR was included in the NMA, but its relatively large sample size may have influenced its SUCRA values. Importantly, pairwise contrasts revealed wide confidence intervals and few statistically significant differences due to small sample sizes and heterogeneous protocols. Hence, while the hierarchy suggests that long-term isometric plantar flexion contraction training and LL-BFR programs are promising for enhancing tendon stiffness, larger, well-controlled trials are needed to confirm these findings and their comparative effectiveness against other loading paradigms.

Notably, most studies measured stiffness under MVC conditions using the slope of the force‒elongation curve. However, there are certain differences in the range of values for AT stiffness across studies. Stiffness within the 0%–25% MVC range is often referred to as passive stiffness, reflecting the inherent material resistance of the tendon to external stretch. In contrast, stiffness measured between 10% and 90% MVC is typically termed active stiffness, representing the load-bearing response of the tendon during muscle contraction and integrating both its viscoelastic properties and neuromuscular force generation. These two constructs have distinct physiological meanings and may respond differently to training and testing conditions. Therefore, pooled stiffness estimates that combine passive- and active-stiffness paradigms should be interpreted with caution, particularly when drawing inferences from pooled effect sizes. Measurement errors in tendon length changes during stiffness assessments are attributed primarily to ultrasound probe misplacement, skin movement, and limitations in tracking displacement (86, 87). Furthermore, stiffness values derived under MVC conditions may not fully capture tendon behaviour during dynamic, functional activities, as these activities are confined to static, isometric testing environments. As a complementary tool, shear wave elastography offers real-time, localized assessments of tendon stiffness under physiological loading, potentially improving its ecological validity (101).

### 4.5 Transitivity assessment and methodological robustness

The validity of the network meta-analysis depends on the transitivity assumption, i.e., the comparability of key effect modifiers across intervention nodes. Although our inclusion criteria restricted participants to healthy adults within a similar age range, baseline physical activity, training status, sex distribution, and intervention dosing/progression were not consistently reported and were not evenly distributed across studies. Such imbalances and reporting gaps may compromise transitivity for certain indirect comparisons and may affect the stability and clinical generalisability of some relative rankings, particularly for sparsely informed nodes. Accordingly, NMA findings and SUCRA rankings should be interpreted alongside effect estimates and their uncertainty, with appropriate caution where transitivity is less plausible.

Because testing paradigms and derivation assumptions varied across trials, outcomes differ in their robustness to methodological heterogeneity. Morphological measures that are directly quantified from imaging (e.g., AT CSA and length), and elongation/strain derived under comparable imaging paradigms, are generally more comparable across studies and were considered relatively robust. In contrast, stiffness pooled across different MVC ranges may reflect partially distinct constructs (passive vs. active stiffness), and derived outcomes such as AT force and especially stress are more sensitive to upstream assumptions, including moment-arm modeling, co-contraction handling, normalization procedures, and the anatomical site used for CSA measurement. Accordingly, stiffness/force/stress findings were interpreted cautiously alongside effect estimates and uncertainty and should be regarded as hypothesis-generating rather than prescriptive.

### 4.6 Limitations

Several limitations should be considered when interpreting the findings of this review. First, intervention and participant heterogeneity were substantial. Fifteen exercise modalities were examined across 49 trials, 88% of which enrolled fewer than 50 participants. Baseline characteristics, e.g., habitual physical activity, training status, age, and sex, were inconsistently reported or controlled, restricting external validity. Second, the outcome assessment lacked uniformity and ecological validity. Diverse imaging and biomechanical techniques (ultrasound, MRI, dynamometry, force plates, ankle ergometers, and shear wave elastography) with variable joint positions, probe pressures, normalization procedures, and data-processing algorithms, all of which may introduce measurement bias. Moreover, few studies have included follow-up testing to determine the durability or functional relevance of the observed adaptations. Third, the limited sample size undermined the statistical power and ranking stability. Wide confidence intervals increased the risk of type II error, and several high SUCRA rankings were driven by imprecise estimates from single studies, yielding putative “winners” that were not corroborated by direct pairwise contrasts. Fourth, there is no consensus on clinically meaningful thresholds (“optimal ranges”) for tendon morphology or mechanical metrics, and anchor-based minimal clinically important differences (MCIDs) are lacking. Fifth, grouping interventions into discrete network nodes assumes a degree of within-node homogeneity. Although different intensity or dosing variants within the same intervention were consolidated using standard Cochrane procedures to improve comparability, residual variability in intensity, weekly volume, progression schemes, and execution details across trials may still obscure within-node effect modifiers, limit dose-specific inference, and reduce ranking stability—particularly in sparsely connected or imprecise nodes. Rankings were therefore interpreted alongside effect estimates and associated uncertainty. Consequently, absolute values are difficult to interpret, and our conclusions necessarily emphasize rank-based inferences from surrogate tendon outcomes rather than prescriptions to maximize any single metric; SUCRA reflects the probability of relative superiority, not effect magnitude, and rankings may be sensitive to modeling choices and between-study heterogeneity. Collectively, these issues highlight the need for adequately powered, head-to-head randomized controlled trials that employ standardized loading definitions, core outcome sets with established MCIDs, and longer-term follow-up linking mechanical changes to symptoms, performance, and injury risk.

## 5 Conclusions

In conclusion, evidence from 49 trials suggests that progressive, higher-dose mechanical loading is most consistently associated with improvements in AT morphology and mechanics in healthy, asymptomatic adults. For prevention-oriented conditioning, gait retraining (5–48 min/session, 3 sessions/week, 12 weeks), isometric plantar flexion contraction (80%–90% MVC, 3–4 sessions/week, 8–14 weeks), and PJT (200–400 contacts/week, 8–12 weeks) ranked among the top options when delivered within these dose–duration ranges. Multimodal strategies, particularly NMES combined with isometric plantar flexion contraction and LL-BFR, further enhance force, stress, and stiffness. However, these findings were supported by a limited number of studies and should be interpreted with caution when considering broader application. By contrast, stretching-dominant programs provide minimal benefit and should not be first-line for tendon adaptation. These findings provide actionable dosing targets and modality choices for enhancing tendon capacity in healthy adults. Future trials should standardize protocols, report exposure precisely, and include clinically anchored endpoints such as injury incidence and time-loss. Importantly, these rankings reflect relative ranking probabilities rather than definitive clinical superiority, and they should be considered hypothesis-generating rather than prescriptive, particularly in healthy but heterogeneous populations.

## Data Availability

Publicly available datasets were analyzed in this study. This data can be found here: the datasets used and/or analyzed during the current study are available from the corresponding author on reasonable request.

## References

[1] Scott SH Winter DA. Internal forces of chronic running injury sites. Med Sci Sports Exerc. (1990) 22(3):357–69. 10.1249/00005768-199006000-00013 2381304

[2] Gross CE Nunley JA 2nd.. Acute achilles tendon ruptures. Foot Ankle Int. (2016) 37(2):233–9. 10.1177/1071100715619606 26590377

[3] de Jonge S van den Berg C de Vos RJ van der Heide HJ Weir A Verhaar JA Incidence of midportion achilles tendinopathy in the general population. Br J Sports Med. (2011) 45(13):1026–8. 10.1136/bjsports-2011-090342 21926076

[4] Nilsson N Nilsson Helander K Hamrin Senorski E Holm A Karlsson J Svensson M The economic cost and patient-reported outcomes of chronic achilles tendon ruptures. J Exp Orthop. (2020) 7(1):60. 10.1186/s40634-020-00277-z 32748273 PMC7399724

[5] Winnicki K Ochała-Kłos A Rutowicz B Pękala PA Tomaszewski KA. Functional anatomy, histology and biomechanics of the human achilles tendon - A comprehensive review. Ann Anat. (2020) 229:151461. 10.1016/j.aanat.2020.151461 31978571

[6] Magnusson SP Kjaer M. The impact of loading, unloading, ageing and injury on the human tendon. J Physiol. (2019) 597(5):1283–98. 10.1113/JP275450 29920664 PMC6395417

[7] Kjaer M. Role of extracellular matrix in adaptation of tendon and skeletal muscle to mechanical loading. Physiol Rev. (2004) 84(2):649–98. 10.1152/physrev.00031.2003 15044685

[8] Pizzolato C Lloyd DG Zheng MH Besier TF Shim VB Obst SJ Finding the sweet spot via personalised achilles tendon training: the future is within reach. Br J Sports Med. (2019) 53(1):11–2. 10.1136/bjsports-2018-099020 30030281

[9] Cooper K Alexander L Brandie D Brown VT Greig L Harrison I Exercise therapy for tendinopathy: a mixed-methods evidence synthesis exploring feasibility, acceptability and effectiveness. Health Technol Assess. (2023) 27(24):1–389. 10.3310/TFWS2748 PMC10641714 37929629

[10] Lazarczuk SL Maniar N Opar DA Duhig SJ Shield A Barrett RS Mechanical, material and morphological adaptations of healthy lower limb tendons to mechanical loading: a systematic review and meta-analysis. Sports Med. (2022) 52(10):2405–29. 10.1007/s40279-022-01695-y 35657492 PMC9474511

[11] Létocart A Grosset JF. Achilles tendon adaptation to neuromuscular electrical stimulation: morphological and mechanical changes. Int J Sports Med. (2021) 42(7):651–61. 10.1055/a-1270-7568 33285575

[12] Centner C Lauber B Seynnes OR Jerger S Sohnius T Gollhofer A Low-load blood flow restriction training induces similar morphological and mechanical achilles tendon adaptations compared with high-load resistance training. J Appl Physiol (1985). (2019) 127(6):1660–7. 10.1152/japplphysiol.00602.2019 31725362

[13] Bohm S Mersmann F Tettke M Kraft M Arampatzis A. Human achilles tendon plasticity in response to cyclic strain: effect of rate and duration. J Exp Biol. (2014) 217(Pt 22):4010–7. 10.1242/jeb.112268 25267851

[14] Zhang Y Xu K Yin M Duan B Kong H Xie Y Effects of blood flow restriction training in athletes: a systematic review and meta-analysis. Int J Sports Med. (2025) 46(7):467–81. 10.1055/a-2537-5879 39933726

[15] Cook SB Scott BR Hayes KL Murphy BG. Neuromuscular adaptations to low-load blood flow restricted resistance training. J Sports Sci Med. (2018) 17(1):66–73. 29535579 PMC5844210

[16] Kubo K Morimoto M Komuro T Yata H Tsunoda N Kanehisa H Effects of plyometric and weight training on muscle-tendon complex and jump performance. Med Sci Sports Exerc. (2007) 39(10):1801–10. 10.1249/mss.0b013e31813e630a 17909408

[17] Centner C Jerger S Lauber B Seynnes O Friedrich T Lolli D Similar patterns of tendon regional hypertrophy after low-load blood flow restriction and high-load resistance training. Scand J Med Sci Sports. (2023) 33(6):848–56. 10.1111/sms.14321 36703264

[18] Heinemeier KM Kjaer M. *In vivo* investigation of tendon responses to mechanical loading. J Musculoskelet Neuronal Interact. (2011) 11(2):115–23. 21625048

[19] Kubo K Ishigaki T Ikebukuro T. Effects of plyometric and isometric training on muscle and tendon stiffness *in vivo*. Physiol Rep. (2017) 5(15):e13374. 10.14814/phy2.13374 28801518 PMC5555899

[20] Bohm S Mersmann F Arampatzis A. Human tendon adaptation in response to mechanical loading: a systematic review and meta-analysis of exercise intervention studies on healthy adults. Sports Med Open. (2015) 1(1):7. 10.1186/s40798-015-0009-9 27747846 PMC4532714

[21] Bucher HC Guyatt GH Griffith LE Walter SD. The results of direct and indirect treatment comparisons in meta-analysis of randomized controlled trials. J Clin Epidemiol. (1997) 50(6):683–91. 10.1016/S0895-4356(97)00049-8 9250266

[22] Ozgundondu B Gok Metin Z. Effects of progressive muscle relaxation combined with music on stress, fatigue, and coping styles among intensive care nurses. Intensive Crit Care Nurs. (2019) 54:54–63. 10.1016/j.iccn.2019.07.007 31371164

[23] Chen R Guo Y Kuang Y Zhang Q. Effects of home-based exercise interventions on post-stroke depression: a systematic review and network meta-analysis. Int J Nurs Stud. (2024) 152:104698. 10.1016/j.ijnurstu.2024.104698 38290424

[24] Bafeta A Trinquart L Seror R Ravaud P. Reporting of results from network meta-analyses: methodological systematic review. Br Med J. (2014) 348:g1741. 10.1136/bmj.g1741 24618053 PMC3949412

[25] Higgins JPT Green S. Cochrane handbook for systematic reviews of interventions Version 5.1. 0. The Cochrane Collaboration; 2011 March. Available online at: https://www.cochrane.org/authors/handbooks-and-manuals/handbook (Accessed February 18, 2025).

[26] Moher D Liberati A Tetzlaff J Altman DG. Preferred reporting items for systematic reviews and meta-analyses: the PRISMA statement. Open Med. (2009) 3(3):e123–30. 10.1371/journal.pmed.1000097 21603045 PMC3090117

[27] Dinh NV Freeman H Granger J Wong S Johanson M. Calf stretching in non-weight bearing versus weight bearing. Int J Sports Med. (2011) 32(3):205–10. 10.1055/s-0030-1268505 21181639

[28] Peres SE Draper DO Knight KL Ricard MD. Pulsed shortwave diathermy and prolonged long-duration stretching increase dorsiflexion range of motion more than identical stretching without diathermy. J Athl Train. (2002) 37(1):43–50. 12937443 PMC164307

[29] Johanson MA Cuda BJ Koontz JE Stell JC Abelew TA. Effect of stretching on ankle and knee angles and gastrocnemius activity during the stance phase of gait. J Sport Rehabil. (2009) 18(4):521–34. 10.1123/jsr.18.4.521 20108853

[30] Mak MK Wong-Yu IS Shen X Chung CL. Long-term effects of exercise and physical therapy in people with Parkinson disease. Nat Rev Neurol. (2017) 13(11):689–703. 10.1038/nrneurol.2017.128 29027544

[31] Adam NC Smith CR Herzog W Amis AA Arampatzis A Taylor WR. *In Vivo* strain patterns in the achilles tendon during dynamic activities: a comprehensive survey of the literature. Sports Med Open. (2023) 9(1):60. 10.1186/s40798-023-00604-5 37466866 PMC10356630

[32] Lopez P Radaelli R Taaffe DR Newton RU Galvão DA Trajano GS Resistance training load effects on muscle hypertrophy and strength gain: systematic review and network meta-analysis. Med Sci Sports Exerc. (2021) 53(6):1206–16. 10.1249/MSS.0000000000002585 33433148 PMC8126497

[33] Burda BU O'Connor EA Webber EM Redmond N Perdue LA. Estimating data from figures with a web-based program: considerations for a systematic review. Res Synth Methods. (2017) 8(3):258–62. 10.1002/jrsm.1232 28268241

[34] Downs SH Black N. The feasibility of creating a checklist for the assessment of the methodological quality both of randomised and non-randomised studies of health care interventions. J Epidemiol Community Health. (1998) 52(6):377–84. 10.1136/jech.52.6.377 9764259 PMC1756728

[35] Almeida MO Davis IS Lopes AD. Biomechanical differences of foot-strike patterns during running: a systematic review with meta-analysis. J Orthop Sports Phys Ther. (2015) 45(10):738–55. 10.2519/jospt.2015.6019 26304644

[36] Urlando A Hawkins D. Achilles tendon adaptation during strength training in young adults. Med Sci Sports Exerc. (2007) 39(7):1147–52. 10.1249/mss.0b013e31805371d1 17596783

[37] Joseph MF Histen K Arntsen J L'Hereux L Defeo C Lockwood D Achilles tendon adaptation during transition to a minimalist running style. J Sport Rehabil. (2017) 26(2):165–70. 10.1123/jsr.2016-0007 27632879

[38] Milgrom Y Milgrom C Altaras T Globus O Zeltzer E Finestone AS. Achilles tendons hypertrophy in response to high loading training. Foot Ankle Int. (2014) 35(12):1303–8. 10.1177/1071100714550651 25212862

[39] Arampatzis A Karamanidis K Albracht K. Adaptational responses of the human achilles tendon by modulation of the applied cyclic strain magnitude. J Exp Biol. (2007) 210(Pt 15):2743–53. 10.1242/jeb.003814 17644689

[40] Deng L Zhang X Xiao S Yang Y Li L Fu W. Changes in the plantar flexion torque of the ankle and in the morphological characteristics and mechanical properties of the achilles tendon after 12-week gait retraining. Life (Basel). (2020) 10(9):159. 10.3390/life10090159 32842586 PMC7555353

[41] Laurent C Baudry S Duchateau J. Comparison of plyometric training with two different jumping techniques on achilles tendon properties and jump performances. J Strength Cond Res. (2020) 34(6):1503–10. 10.1519/JSC.0000000000003604 32271290

[42] Kay AD Blazevich AJ Tysoe JC Baxter BA. Cross-Education effects of isokinetic eccentric plantarflexor training on flexibility, strength, and muscle-tendon mechanics. Med Sci Sports Exerc. (2024) 56(7):1242–55. 10.1249/MSS.0000000000003418 38451696

[43] Peixinho CC Silva GA Brandao MCA Menegaldo LL de Oliveira LF. Effect of a 10-week stretching program of the Triceps surae muscle architecture and tendon mechanical properties. J Sci Sport Exerc. (2021) 3(2):107–14. 10.1007/s42978-021-00110-8

[44] Hansen P Aagaard P Kjaer M Larsson B Magnusson SP. Effect of habitual running on human achilles tendon load-deformation properties and cross-sectional area. J Appl Physiol (1985). (2003) 95(6):2375–80. 10.1152/japplphysiol.00503.2003 12937029

[45] Konrad A Gad M Tilp M. Effect of PNF stretching training on the properties of human muscle and tendon structures. Scand J Med Sci Sports. (2015) 25(3):346–55. 10.1111/sms.12228 24716522

[46] Mahieu NN Cools A De Wilde B Boon M Witvrouw E. Effect of proprioceptive neuromuscular facilitation stretching on the plantar flexor muscle-tendon tissue properties. Scand J Med Sci Sports. (2009) 19(4):553–60. 10.1111/j.1600-0838.2008.00815.x 18627559

[47] Mahieu NN McNair P De Muynck M Stevens V Blanckaert I Smits N Effect of static and ballistic stretching on the muscle-tendon tissue properties. Med Sci Sports Exerc. (2007) 39(3):494–501. 10.1249/01.mss.0000247004.40212.f7 17473776

[48] Tsai MS Domroes T Pentidis N Koschinski S Schroll A Bohm S Effect of the temporal coordination and volume of cyclic mechanical loading on human achilles tendon adaptation in men. Sci Rep. (2024) 14(1):6875. 10.1038/s41598-024-56840-6 38519507 PMC10960029

[49] Werkhausen A Albracht K Cronin NJ Paulsen G Bojsen-Møller J Seynnes OR. Effect of training-induced changes in achilles tendon stiffness on muscle-tendon behavior during landing. Front Physiol. (2018) 9:794. 10.3389/fphys.2018.00794 29997526 PMC6028711

[50] Zhang X Deng L Yang Y Xiao S Li L Fu W. Effects of 12-week transition training with minimalist shoes on achilles tendon loading in habitual rearfoot strike runners. J Biomech. (2021) 128:110807. 10.1016/j.jbiomech.2021.110807 34670150

[51] Zhang X Deng L Xiao S Fu W. Effects of a 12-week gait retraining program on the achilles tendon adaptation of habitually shod runners. Scand J Med Sci Sports. (2024) 34(1):e14516. 10.1111/sms.14516 37817483

[52] Konrad A Tilp M. Effects of ballistic stretching training on the properties of human muscle and tendon structures. J Appl Physiol (1985). (2014) 117(1):29–35. 10.1152/japplphysiol.00195.2014 24812641

[53] Morrissey D Roskilly A Twycross-Lewis R Isinkaye T Screen H Woledge R The effect of eccentric and concentric calf muscle training on achilles tendon stiffness. Clin Rehabil. (2011) 25(3):238–47. 10.1177/0269215510382600 20980351

[54] Houghton LA Dawson BT Rubenson J. Effects of plyometric training on achilles tendon properties and shuttle running during a simulated cricket batting innings. J Strength Cond Res. (2013) 27(4):1036–46. 10.1519/JSC.0b013e3182651e7a 22739327

[55] Fouré A Nordez A Cornu C. Effects of plyometric training on passive stiffness of gastrocnemii muscles and achilles tendon. Eur J Appl Physiol. (2012) 112(8):2849–57. 10.1007/s00421-011-2256-x 22131086

[56] Fouré A Nordez A Guette M Cornu C. Effects of plyometric training on passive stiffness of gastrocnemii and the musculo-articular complex of the ankle joint. Scand J Med Sci Sports. (2009) 19(6):811–8. 10.1111/j.1600-0838.2008.00853.x 19508650

[57] Jerger S Centner C Lauber B Seynnes O Sohnius T Jendricke P Effects of specific collagen peptide supplementation combined with resistance training on achilles tendon properties. Scand J Med Sci Sports. (2022) 32(7):1131–41. 10.1111/sms.14164 35403756

[58] Ishigaki T Kubo K. Effects of eccentric training with different training frequencies on blood circulation, collagen fiber orientation, and mechanical properties of human achilles tendons *in vivo*. Eur J Appl Physiol. (2018) 118(12):2617–26. 10.1007/s00421-018-3985-x 30203295

[59] Geremia JM Baroni BM Bobbert MF Bini RR Lanferdini FJ Vaz MA. Effects of high loading by eccentric triceps surae training on achilles tendon properties in humans. Eur J Appl Physiol. (2018) 118(8):1725–36. 10.1007/s00421-018-3904-1 29858689

[60] Cini A Sonda FC da Rocha ES Borges M Felappi CJ Vaz MA Effects of passive static stretching on the achilles tendon properties of adults: a randomized controlled trial. Sport Sci Health. (2024) 20(2):337–46. 10.1007/s11332-023-01119-y

[61] Bohm S Mersmann F Santuz A Arampatzis A. Enthalpy efficiency of the soleus muscle contributes to improvements in running economy. Proc Biol Sci. (2021) 288(1943):20202784. 10.1098/rspb.2020.2784 33499791 PMC7893283

[62] Albracht K Arampatzis A. Exercise-induced changes in triceps surae tendon stiffness and muscle strength affect running economy in humans. Eur J Appl Physiol. (2013) 113(6):1605–15. 10.1007/s00421-012-2585-4 23328797

[63] Waugh CM Alktebi T de Sa A Scott A. Impact of rest duration on achilles tendon structure and function following isometric training. Scand J Med Sci Sports. (2018) 28(2):436–45. 10.1111/sms.12930 28603874

[64] Konrad A Tilp M. Increased range of motion after static stretching is not due to changes in muscle and tendon structures. Clin Biomech. (2014) 29(6):636–42. 10.1016/j.clinbiomech.2014.04.013 24856792

[65] Arampatzis A Peper A Bierbaum S Albracht K. Plasticity of human achilles tendon mechanical and morphological properties in response to cyclic strain. J Biomech. (2010) 43(16):3073–9. 10.1016/j.jbiomech.2010.08.014 20863501

[66] Fouré A Nordez A Cornu C. Plyometric training effects on achilles tendon stiffness and dissipative properties. J Appl Physiol (1985). (2010) 109(3):849–54. 10.1152/japplphysiol.01150.2009 20576842

[67] Hirayama K Iwanuma S Ikeda N Yoshikawa A Ema R Kawakami Y. Plyometric training favors optimizing muscle-tendon behavior during depth jumping. Front Physiol. (2017) 8:16. 10.3389/fphys.2017.00016 28179885 PMC5263151

[68] Wu YK Lien YH Lin KH Shih TT Wang TG Wang HK. Relationships between three potentiation effects of plyometric training and performance. Scand J Med Sci Sports. (2010) 20(1):e80–6. 10.1111/j.1600-0838.2009.00908.x 19486477

[69] Simpson CL Kim BDH Bourcet MR Jones GR Jakobi JM. Stretch training induces unequal adaptation in muscle fascicles and thickness in medial and lateral gastrocnemii. Scand J Med Sci Sports. (2017) 27(12):1597–604. 10.1111/sms.12822 28138986

[70] Létocart AJ Svensson RB Mabesoone F Charleux F Marin F Dermigny Q Structure and function of achilles and patellar tendons following moderate slow resistance training in young and old men. Eur J Appl Physiol. (2024) 124(9):2707–23. 10.1007/s00421-024-05461-y 38649478

[71] Waugh CM Scott A. Substantial achilles adaptation following strength training has no impact on tendon function during walking. PLoS One. (2021) 16(7):e0255221. 10.1371/journal.pone.0255221 34324575 PMC8320898

[72] Neves CD Sponbeck JK Neves KA Mitchell UH Hunter I Johnson AW. The achilles tendon response to a bout of running is not affected by Triceps surae stretch training in runners. J Sports Sci Med. (2020) 19(2):358–63. 32390729 PMC7196739

[73] Fouré A Nordez A Cornu C. Effects of eccentric training on mechanical properties of the plantar flexor muscle-tendon complex. J Appl Physiol (1985). (2013) 114(5):523–37. 10.1152/japplphysiol.01313.2011 23239873

[74] Kubo K Ikebukuro T Maki A Yata H Tsunoda N. Time course of changes in the human achilles tendon properties and metabolism during training and detraining *in vivo*. Eur J Appl Physiol. (2012) 112(7):2679–91. 10.1007/s00421-011-2248-x 22105708

[75] Ikeda N Hiratsuka K Isaka T. Effect of 6-week instrument-assisted soft tissue mobilization on joint flexibility and musculotendinous properties. Sports (Basel). (2024) 12(6):150. 10.3390/sports12060150 38921844 PMC11209483

[76] Mahieu NN McNair P Cools A D'Haen C Vandermeulen K Witvrouw E. Effect of eccentric training on the plantar flexor muscle-tendon tissue properties. Med Sci Sports Exerc. (2008) 40(1):117–23. 10.1249/mss.0b013e3181599254 18091014

[77] Fouré A Nordez A McNair P Cornu C. Effects of plyometric training on both active and passive parts of the plantarflexors series elastic component stiffness of muscle-tendon complex. Eur J Appl Physiol. (2011) 111(3):539–48. 10.1007/s00421-010-1667-4 20931220

[78] Torres-Banduc M Chirosa-Ríos L Chirosa-Rios I Jerez-Mayorga D. Minimum effective plyometric training volume in sedentary individuals: impact on lower limb viscoelastic properties and functional performance in a randomised controlled trial. J Sports Sci. (2024) 42(19):1794–805. 10.1080/02640414.2024.2413327 39390626

[79] Werkhausen A Cronin NJ Albracht K Paulsen G Larsen AV Bojsen-Møller J Training-induced increase in achilles tendon stiffness affects tendon strain pattern during running. PeerJ. (2019) 7:e6764. 10.7717/peerj.6764 31086731 PMC6486809

[80] Sanz-López F Berzosa Sánchez C Hita-Contreras F Cruz-Diaz D Martínez-Amat A. Ultrasound changes in achilles tendon and gastrocnemius Medialis muscle on squat eccentric overload and running performance. J Strength Cond Res. (2016) 30(7):2010–8. 10.1519/JSC.0000000000001298 26677829

[81] Ahmad Z Wardale J Brooks R Henson F Noorani A Rushton N. Exploring the application of stem cells in tendon repair and regeneration. Arthroscopy. (2012) 28(7):1018–29. 10.1016/j.arthro.2011.12.009 22381688

[82] Woo SL Gomez MA Woo YK Akeson WH. Mechanical properties of tendons and ligaments. II. The relationships of immobilization and exercise on tissue remodeling. Biorheology. (1982) 19(3):397–408. 10.3233/bir-1982-19302 7104481

[83] Yao W Zhang Y Zhang L Zhou J Zhang Y Zheng X MRI Features of and factors related to ankle injuries in asymptomatic amateur marathon runners. Skeletal Radiol. (2021) 50(1):87–95. 10.1007/s00256-020-03530-9 32632469 PMC7677287

[84] Kharazi M Bohm S Theodorakis C Mersmann F Arampatzis A. Quantifying mechanical loading and elastic strain energy of the human achilles tendon during walking and running. Sci Rep. (2021) 11(1):5830. 10.1038/s41598-021-84847-w 33712639 PMC7955091

[85] Massimo SS Fabio V Al Khayyat SG Danilo D Barbara C Marco B Ultrasonographic insights into the complex anatomy and biomechanical dynamics of the achilles tendon and its fascicles: a pictorial essay. J Ultrasound. (2025) 28(2):505–16. 10.1007/s40477-025-00987-z 39899233 PMC12145380

[86] Castro J Livino de Carvalho K Silva PE Fachin-Martins E Babault N Marqueti RC Intra- and inter-rater reproducibility of ultrasound imaging of patellar and quadriceps tendons in critically ill patients. PLoS One. (2019) 14(6):e0219057. 10.1371/journal.pone.0219057 31247020 PMC6597100

[87] Lunardi M Furtado FE Sakugawa RL Sonda FC Sampaio LT Diefenthaeler F. Reliability of a special device for measuring the cross-sectional area of the patellar tendon by ultrasonography. J Ultrasound. (2023) 26(4):897–903. 10.1007/s40477-023-00829-w 37743436 PMC10632332

[88] Hager B Schreiner MM Walzer SM Hirtler L Mlynarik V Berg A Transverse relaxation anisotropy of the achilles and patellar tendon studied by MR microscopy. J Magn Reson Imaging. (2022) 56(4):1091–103. 10.1002/jmri.28095 35122454 PMC9545006

[89] Du J Pak BC Znamirowski R Statum S Takahashi A Chung CB Magic angle effect in magnetic resonance imaging of the achilles tendon and enthesis. Magn Reson Imaging. (2009) 27(4):557–64. 10.1016/j.mri.2008.09.003 19022600

[90] Marshall H Howarth C Larkman DJ Herlihy AH Oatridge A Bydder GM. Contrast-enhanced magic-angle MR imaging of the achilles tendon. AJR Am J Roentgenol. (2002) 179(1):187–92. 10.2214/ajr.179.1.1790187 12076933

[91] Reeves ND Cooper G. Is human achilles tendon deformation greater in regions where cross-sectional area is smaller? J Exp Biol. (2017) 220(Pt 9):1634–42. 10.1242/jeb.157289 28202585

[92] Magnusson SP Kjaer M. Region-specific differences in achilles tendon cross-sectional area in runners and non-runners. Eur J Appl Physiol. (2003) 90(5-6):549–53. 10.1007/s00421-003-0865-8 12905044

[93] Jiang D Jiang Z Zhang Y Wang S Yang S Xu B Effect of young extrinsic environment stimulated by hypoxia on the function of aged tendon stem cell. Cell Biochem Biophys. (2014) 70(2):967–73. 10.1007/s12013-014-0004-7 24817591

[94] Shill DD Polley KR Willingham TB Call JA Murrow JR McCully KK Experimental intermittent ischemia augments exercise-induced inflammatory cytokine production. J Appl Physiol (1985). (2017) 123(2):434–41. 10.1152/japplphysiol.01006.2016 28572502

[95] Jia YY Zhou JY Chang Y An F Li XW Xu XY Effect of optimized concentrations of basic fibroblast growth factor and epidermal growth factor on proliferation of fibroblasts and expression of collagen: related to pelvic floor tissue regeneration. Chin Med J (Engl). (2018) 131(17):2089–96. 10.4103/0366-6999.239301 30127219 PMC6111681

[96] Makino T Jinnin M Muchemwa FC Fukushima S Kogushi-Nishi H Moriya C Basic fibroblast growth factor stimulates the proliferation of human dermal fibroblasts via the ERK1/2 and JNK pathways. Br J Dermatol. (2010) 162(4):717–23. 10.1111/j.1365-2133.2009.09581.x 19995368

[97] Yang G Crawford RC Wang JH. Proliferation and collagen production of human patellar tendon fibroblasts in response to cyclic uniaxial stretching in serum-free conditions. J Biomech. (2004) 37(10):1543–50. 10.1016/j.jbiomech.2004.01.005 15336929

[98] Connizzo BK Yannascoli SM Soslowsky LJ. Structure-function relationships of postnatal tendon development: a parallel to healing. Matrix Biol. (2013) 32(2):106–16. 10.1016/j.matbio.2013.01.007 23357642 PMC3615149

[99] Rigozzi S Müller R Stemmer A Snedeker JG. Tendon glycosaminoglycan proteoglycan sidechains promote collagen fibril sliding-AFM observations at the nanoscale. J Biomech. (2013) 46(4):813–8. 10.1016/j.jbiomech.2012.11.017 23219277

[100] Connizzo BK Sarver JJ Birk DE Soslowsky LJ Iozzo RV. Effect of age and proteoglycan deficiency on collagen fiber re-alignment and mechanical properties in mouse supraspinatus tendon. J Biomech Eng. (2013) 135(2):021019. 10.1115/1.4023234 23445064 PMC5413158

[101] Mifsud T Chatzistergos P Maganaris C Chockalingam N Padhiar N Stafrace KM Supersonic shear wave elastography of human tendons is associated with *in vivo* tendon stiffness over small strains. J Biomech. (2023) 152:111558. 10.1016/j.jbiomech.2023.111558 37004390

