# Supplementary material for: The effect of exercise training modalities on the morphological and mechanical properties of the achilles tendon: a systematic review and network meta-analysis
Source: Front Sports Act Living. 2026 Mar 12;8:1782503. doi: 10.3389/fspor.2026.1782503 (PMC13017926; doi:10.3389/fspor.2026.1782503)
Supplement: Supplementary file 1 [file Datasheet1.docx]

Supplementary Material

Supplementary Table S1 Methods of risk of bias assessment

| **Criteria** | **Description** |
| --- | --- |
| **1. Aim clearly described** | The aim/hypothesis/objective is clearly described. |
| **2. Outcomes described** | The main outcomes to be measured are clearly described in the introduction or methods section. |
| **3. Subjects clearly described** | The characteristics of the subjects included in the trial are clearly described. |
| **4. Interventions clearly described** | Each intervention to be compared is clearly described. |
| **5. Distribution of confounders described** | Confounding factors are clearly described. Confounding factors are clearly described. Confounders to be considered include subject’s sex, weight, fitness level. |
| **6. Main findings clearly described** | Simple outcome data are reported for all major findings so the reader can check the major analysis and conclusions. |
| **7. Estimates of random variability in data** | For nonnormally distributed data, the interquartile range of results should be reported. For normally distributed data, standard deviations or confidence intervals should be reported. |
| **8. All important adverse events reported** | The study demonstrates a comprehensive attempt to record all adverse events. This could include discomfort associated with any condition or delayed-onset muscle soreness. |
| **9. Actual probability values reported** | Actual probability values (e.g., not *P*<.05) have been reported for the main outcomes, except where the probability value is less than .001. |
| **10. Subjects asked are representative of population** | The source population for subjects and how they were selected are described. Subjects would be representative if they comprised the entire population, an unselected sample of consecutive subjects, or a random sample. If the study does not report the proportion of the source population from which the subjects are derived, the answer is no. |
| **11. Subjects representative of population** | The subjects prepared to participate are representative of the entire population from which they were recruited. Validation that the sample was representative would include demonstrating that the distribution of confounding factors was the same in the study sample and the source population. |
| **12. Examiners blinded** | There was an attempt to blind those measuring the main outcomes. |
| **13. Data dredging** | Any analysis that had not been planned at the outset of the study is clearly described. If no retrospective unplanned subgroup analysis is reported, the answer is yes. |
| **14. Appropriate statistical tests** | The statistical tests used to assess the main outcomes are appropriate. |
| **15. Internal validity** | It is necessary for the study subjects to be familiar with the content of the training to be performed and to standardize the movements as much as possible. If the subject is scheduled for familiarization training, the answer is yes. |
| **16. Valid and reliable main outcome measures** | For studies where the outcome measures are clearly described, the answer is yes. For studies that refer to other work or that demonstrate the outcome measures are accurate, the answer is yes. |
| **17. Subjects recruited from same population** | The subjects in different intervention groups were included from the same population. If the subjects acted as their own control, this was answered yes. |
| **18. Subjects recruited over same period** | The subjects in different intervention groups were recruited over the same period. If the subjects acted as their own control, this was answered yes. |
| **19. Subjects randomised to intervention groups** | Studies which state that subjects were randomised should be answered yes except where method of randomisation would not ensure random allocation. For example, alternate allocation would be scored as no because it is predictable. |
| **20. Intervention order randomized** | The order of the intervention tested was randomized. |
| **21. Adequate adjustment for confounding** | There was adequate adjustment for confounding in the analysis from which the main findings were drawn. If the effect of the main confounders was not investigated or confounding was demonstrated but no adjustment was made in the final analysis, this was answered no. |
| **22. Sufficient power** | If the study reported a power calculation, this was answered yes. |

Supplementary Table S2 Participant and stretching training intervention characteristics of the included studies

| **Author** | **Year** | **N (M, F)** | **Population** | **Type** | **Duration (weeks),  Frequency  (p/wk×n)** | **Repeat times** | **Load duration (s)** | **Intensity** | **Rest between sets (s)** | **Movement description** |
| --- | --- | --- | --- | --- | --- | --- | --- | --- | --- | --- |
| Cini et al. [59] | 2024 | 30 (6 M, 24 F) | Healthy people | SST | 6, 3 | - | ①120;  ②300 | - | - | Stand on a 15-cm-high step, supporting with the forefoot, with maximal dorsiflexion. |
| Konrad et al. [44] | 2015 | 49 (31 M, 18 F) | Healthy Police cadets | PNF | 6, 5 | 4 | 144 | - | 0 | 15-s static calf stretch → 6-s isometric contraction of the stretched muscle → 15-s resisted dorsiflexion contraction. |
| Konrad and Tilp [51] | 2014 | 48 (30 M, 18 F) | Police cadets | BST | 6, 5 | 4 | 30 | Stretch to the point of discomfort | 0 | Perform plantar flexor stretching in a standing wall-push position, moving up and down with the front knee at a frequency of 1 Hz. |
| Konrad and Tilp [63] | 2014 | 49 (35 M, 14 F) | Healthy police cadets | SST | 6, 5×4 | 1 | 30 | Stretch to the point of discomfort | 0 | Perform plantar flexor stretching in a standing wall-push position. |
| Mahieu et al. [46] | 2007 | 81 (37 M, 44 F) | - | SST, BST | 6, 7 | 5 | 20 | Stretch to the point of discomfort | 20 | The SST maintains full extension of the back knee, the BST moves the front knee up and down at a rate of once per second. |
| Mahieu et al. [45] | 2009 | 62 (33 M, 29 F) | Recreational athletes | PNF | 6, 7×5 | 1 | 36 | Stretch to the point of discomfort | 20 | 15-s static calf stretch → 6-s isometric contraction of the stretched muscle → 15-s resisted dorsiflexion contraction. |
| Neves et al. [71] | 2020 | 33 (17 M, 16 F) | Recreational runners | SST | 3, 6×2 | 3 | 30 | Stretch to the point of discomfort | - | 1. Keeping the knee straight (gastrocnemius stretch);  2. Slightly bending the knee (soleus stretch). |
| Peixinho et al. [42] | 2021 | 20 (20 M) | Healthy people | SST | 10, 4~5 | 2 | 30 | Stretch to the point of discomfort | 0 | 1. Place the heel on a 30-cm step, keeping the knee fully extended; 2. Lean forwards, reaching for the ground with one leg extended straight back and the other leg bent. |
| Simpson et al. [68] | 2017 | 21 (21 M) | - | SST | 6, 5 | - | 180 | Rotate the ankle to maximum dorsiflexion | - | Use the leg press machine to relax the plantar flexors, rotating the ankle to maximum dorsiflexion. |

M, male; F, female; p/wk, sessions per week; n, number of sessions; SST, static stretching training; PNF, proprioceptive neuromuscular facilitation stretching; BST, ballistic stretching training; -, not mentioned in the study; ①, group 1; ②, group 2.

Supplementary Table S3 Participant and gait retraining intervention characteristics of the included studies

| **Author** | **Year** | **N (M, F)** | **Population** | **Type** | **Duration (weeks), Frequency (p/wk×n)** | **Movement description** |
| --- | --- | --- | --- | --- | --- | --- |
| Deng et al. [39] | 2020 | 34 (20 M, 14 F) | Recreational runners | GR | 12, 3 | Forefoot running in minimalist shoes; optional foot core exercises. |
| Joseph et al. [36] | 2017 | 22 (15 F, 7 M) | Well-trained runners | GR | 12, - | Running with forefoot strike in minimalist shoes. |
| Zhang et al. [49] | 2021 | 17 (17 M) | Recreational runners | GR | 12, 3 | Forefoot running in minimalist shoes; optional foot core exercises. |
| Zhang et al. [50] | 2024 | 28 (28 M) | Recreational runners | GR | 12, 3 | Forefoot running in minimalist shoes; optional foot core exercises. |

M, male; F, female; p/wk, sessions per week; n, number of sessions; GR, gait retraining; -, not mentioned in the study.

Supplementary Table S4 Participant and strength training intervention characteristics of the included studies

| **Author** | **Year** | **N (M, F)** | **Population** | **Type** | **Duration (weeks),  Frequency  (p/wk×n)** | **Repeat times** | **Load duration (s)** | **Intensity** | **Rest between different movements (s)** | **Rest between sets (s)** | **Movement description** |
| --- | --- | --- | --- | --- | --- | --- | --- | --- | --- | --- | --- |
| Albracht and Arampatzis [61] | 2013 | 26 (-) | Recreational long-distance runners | LIPFC | 14, 4×5 | 4 | 3 s loading, 3 s relax | 90% MVC | - | - | Isometric plantar flexion contraction. |
| Arampatzis et al. [38] | 2007 | 21 (7 M, 14 F) | Healthy university adults | LIPFC | 14, 4×5 | ①7; ②4 | 3 s loading, 3 s relax | ①55% MVC; ②90% MVC | - | - | Isometric plantar flexion contraction. |
| Arampatzis et al. [64] | 2010 | 11 (11 M) | Healthy university adults | LIPFC | 14, 4×5 | ①20; ②12 | 1 s loading, 1 s relax | ①55% MVC; ②90% MVC | - | - | Isometric plantar flexion contraction. |
| Bohm et al. [13] | 2014 | 39 (39 M) | Active healthy people | LIPFC | 14, 4×5 | - | ①3 s loading, 3 s relax; ②72 single-leg jumps; ③12 s loading | - | - | - | Isometric contraction in leg press. |
| Bohm et al. [60] | 2021 | 23 (12 M, 11 F) | Recreational runners | LIPFC | 14, (3-4) × 5 | 4 | 3 s loading, 3 s relax | - | - | - | Isometric plantar flexion contraction. |
| Kubo et al. [73] | 2012 | 16 (16 M) | active healthy people | SIPFC | 12, 4 | 15 | 15 | 80% MVC | - | 30 | Unilateral Isometric plantar flexion contraction. |
| Létocart et al. [69] | 2024 | 23 (23 M) | Sedentary young and elderly men | SIPFC | 12,  ①3 × (3-4);  ② 3 × 5 | ①7-15; ②6 | 3 s loading, 3 s relax | 55% 1RM | - | 180 | Training the triceps surae and quadriceps muscles using calf extension, leg extension machines, and seated leg press. |
| Morrissey et al. [52] | 2011 | 38 (14 M, 24 F) | Recreational athlete | SIPFC | 6 | 15 | 2 | - | - | - | Eccentric group: heel drop; Concentric group: heel raise. |
| Tsai et al. [47] | 2024 | 52 (52 M) | Healthy young males | LIPFC | 16, ①4×5; ②2.5×6; ③2.5×10; ④5×3; ⑤5×5 | 12 | 3 s loading, 3 s relax | ①90% MVC=240 s/week; ②180 s/week; ③300 s/week; ④180 s/week; ⑤300 s/week | - | 120 | Isometric plantar flexion contraction. |
| Urlando and Hawkins [35] | 2007 | 10 (10 M) | - | SIPFC | 8, 3×3 | 10 | - | 70% maximum force | - | - | Train using machines that allow them to sit down and perform heel raises. |
| Waugh et al. [62] | 2018 | 14 (7 M, 7 F) | Active healthy people | SIPFC | 12, 3×5 | 10 | 3 | 90% MVC | ①3; ②10 | 90 | Perform plantar flexion contraction training on a isokinetic dynamometer. |
| Waugh and Scott [70] | 2021 | 14 (7 M, 7 F) | - | SIPFC | 12, 3×5 | 10 | 3 | 90% MVC | ①3; ②10 | 90 | Perform plantar flexion contraction training on a isokinetic dynamometer. |
| Werkhausen et al. [48] | 2018 | 21 (11 M, 10 F) | Recreationally active volunteers | SIPFC | 10, 3×4 | 10 | 1 s loading, 5 s relax | 80% maximum force | - | - | Standing isometric unilateral plantar flexion with short-duration explosive contraction. |
| Werkhausen et al. [78] | 2019 | 21 (11 M, 10 F) | Recreationally active adults | SIPFC | 10, 3×4 | 10 | Explosive fixed-end contraction, held for 1 s | 80% maximum force | 5 | - | Plantar flexion contraction training on a custom drilling platform. |
| Fouré et al. [54] | 2012 | 19 (19 M) | - | LPJT | 14, 2-3 | - | - | 1 h (200~600 jumps) |  | - | SJ, CMJ, DJ, a series of jumps over hedges using alternatively one foot or both feet. |
| Fouré et al. [76] | 2011 | 19 (19 M) | - | LPJT | 14, 2-3 | - | - | 1 h (200~600 jumps) |  | - | SJ, CMJ, DJ, a series of jumps over hedges using alternatively one foot or both feet. |
| Fouré et al. [65] | 2010 | 19 (19 M) | - | LPJT | 14, 2-3 | - | - | 1 h (200~600 jumps) |  | - | SJ, CMJ, DJ, a series of jumps over hedges using alternatively one foot or both feet. |
| Fouré et al. [55] | 2009 | 17 (17 M) | - | SPJT | 8, 2 | - | - | 1 h (150~280 jumps) |  | - | SJ, CMJ, DJ, a series of jumps over hedges using alternatively one foot or both feet. |
| Hirayama et al. [66] | 2017 | 21 (21 M) | Active people | SPJT | 12, 3×10 | 10 | - | - |  | 30 | Depth jumps on a sled device from a certain height, involving unidirectional single-joint plantar flexion. |
| Houghton et al. [53] | 2013 | 15 (-) | Healthy cricket player | SPJT | 8, 2 × (8-15) | - | - | 180-300 jumps/week |  | - | Training focus arranged for Stationary hopping, DJ on the spot, and DJ with displacement based on the following: Landing technique, Maximize jump height, Repeated jump. |
| Laurent et al. [40] | 2020 | 32 (17 M, 15 F) | Active students | SPJT | 10, 2 × (3-5) | 10 | - | Week1 200 jumps, +20 jumps/week |  | - | ① knees extended; ② knees to~80–90°Stationary hopping, DJ on the spot and DJ with displacement. |
| Torres-Banduc et al. [77] | 2024 | 44 (22 M, 22 F) | Inactive and sedentary people | SPJT | 4, 3 × (2-5) | 6-20 | - | - |  | 120 | Lateral/Directional Hops, Standing Jumps, Vertical/Explosive Jumps, Single leg bounding. |
| Wu et al. [67] | 2010 | 21 (21 M) | student | SPJT | 8, 2 ×6 | 10 | - | 120-240 jumps/week |  | 30 | Squat jump, Tuck Jump, Double Leg Hop, Box Jump. |
| Fouré et al. [72] | 2013 | 24 (24 M) | Healthy people | LECT | 14, 2-3 | - | - | 1 h 200~600 times | - | - | Eccentric contractions at different heights, performed single- or double-legged; right leg eccentric, left leg concentric. |
| Geremia et al. [58] | 2018 | 15 (15 M) | Recreationally active people | SECT | 12, (1-2) × (3-5) | 10 | - | - | - | 60 | Maximally resist isokinetic dorsiflexion induced by the dynamometer. |
| Ishigaki and Kubo [57] | 2018 | 10 (10 M) | Healthy people | SECT | 12, ①3×6; ②6×6 | 15 | - | - | - | - | Knee fully extended and slightly flexed. |
| Kay et al. [41] | 2024 | 26 (10 M, 16 F) | Recreationally active people | SECT | 6, 2×5 | 10 | 1 | ①50% MVC; ②100% MVC | - | 60 | Repeated eccentric plantar flexion contractions on an isokinetic dynamometer. |
| Mahieu et al. [75] | 2008 | 64 (32 M, 32 F) | Healthy people | SECT | 6, 7×3 | 15 | 6 | - | - | 20 | Single-leg heel drop. |
| Sanz-López et al. [79] | 2016 | 20 (20 M) | Healthy people | SECT | 6, 2×4 | 7 | - | 80% 1RM | - | 120 | Squat training using an isoinertial YoYo squat device. |

M, male; F, female; p/wk, sessions per week; n, number of sessions; LIPFC, long-term isometric plantar flexion contraction; MVC, maximum voluntary contraction; SIPFC, short-term isometric plantar flexion contraction; RM, repetition maximum; SJ, squat jump; CMJ, vertical countermovement jump; DJ, drop jump; LPJT, long-term plyometric jump training; SPJT, short-term plyometric jump training; LECT, long-term eccentric contraction training; SECT, short-term eccentric contraction training; -, not mentioned in the study; ①, group 1; ②, group 2; ③, group 3; ④, group 4; ⑤, group 5; YoYo, a flywheel technology.

Supplementary Table S5 Participant and multimodal training intervention characteristics of the included studies

| **Author** | **Year** | **N (M, F)** | **Population** | **Type** | **Duration (weeks), Frequency (p/wk×n)** | **Repeat times** | **Load duration (s)** | **Intensity** | **Rest between sets (s)** | **Movement description** |
| --- | --- | --- | --- | --- | --- | --- | --- | --- | --- | --- |
| Centner et al. [12] | 2019 | 55 (55 M) | Healthy people | LL-BFR,  LIPFC | 14, ① (2-3) ×3; ②14, (2-3) ×4 | - | - | ①70%~85% 1RM;  ②20% 1RM | 60 | Sitting and standing isometric plantar flexion contraction. |
| Centner et al. [17] | 2023 | 29 (29 M) | - | LL-BFR,  LIPFC | 14, ①3×3;  ②3×4 | ① Start with 12 reps, decreasing by 2 reps/set  ② First set 30 reps, remaining 15 reps/set | - | ①70%~85% 1RM (+5% 1RM/week)  ②20%~35% 1RM (+5% 1RM/week) | 60 | Sitting and standing isometric plantar flexion contraction. |
| Ikeda et al. [74] | 2024 | 14 (14 M) | Recreational player | IASTM | 6, 3 | - | ① 60 entire calf posterior; ② 90 gastrocnemius and the tendon junction near the gastrocnemius and AT; ③ 45 soleus; ④ 60 AT; ⑤ 45 tibialis posterior, flexor hallucis longus, and flexor digitorum longus | - | - | Generate gentle tapping, apply compressive and shear stresses, and induce tensile forces in the engaged tissues. |
| Jerger et al. [56] | 2022 | 27 (27 M) | Healthy people | LIPFC,  LIPFC＋SCP | 14, 3×3 | 12-6 | - | 70%~85% 1RM | 60 | Sitting and standing isometric plantar flexion contraction. |
| Létocart and Grosset [11] | 2021 | 8 (8 M) | - | NMES+IPFC | 12, 3 | 45 | 6.25 | Until reaching the maximum tolerable intensity (pain threshold) | - | Isometric plantar flexion contraction. |

M, male; F, female; p/wk, sessions per week; n, number of sessions; LL-BFR, low-load blood ﬂow restriction training; LIPFC, long-term isometric plantar flexion contraction; RM, repetition maximum; rep, repetition; IASTM, instrument-assisted soft tissue mobilization; AT, Achilles tendon; IPFC + SCP, specific collagen peptide supplementation combined with resistance training; NMES+IPFC, neuromuscular electrical stimulation with isometric plantar flexion contraction; -, not mentioned in the study; ①, group 1; ②, group 2; ③, group 3; ④, group 4; ⑤, group 5.

Supplementary Table S6 Participant and endurance training intervention characteristics of the included studies

| **Author** | **Year** | **N (M, F)** | **Population** | **Type** | **Duration (weeks),**  **Frequency (p/wk×n)** | **Load duration(s)** | **Intensity** | **Movement description** |
| --- | --- | --- | --- | --- | --- | --- | --- | --- |
| Hansen et al. [43] | 2003 | 11 (7 M, 4 F) | Healthy people | ET | 36, 2-3 | 1800~3000 | 9 months 70~80 times | Habitual running training. |
| Milgrom et al. [37] | 2014 | 55 (-) | New elite infantry recruits | ET | 24, - | - | - | Basic infantry training. |

M, male; F, female; p/wk, sessions per week; n, number of sessions; ET, endurance training; -, not mentioned in the study.

Supplementary Table S7 Results of the risk-of-bias assessment of the included studies

| **Study** | **Year** | **Item*** | | | | | | | | | | | | | | | | | | | | | | **Total** | | **Risk** |
| --- | --- | --- | --- | --- | --- | --- | --- | --- | --- | --- | --- | --- | --- | --- | --- | --- | --- | --- | --- | --- | --- | --- | --- | --- | --- | --- |
|  |  | **1** | **2** | **3** | **4** | **5** | **6** | **7** | **8** | **9** | **10** | **11** | **12** | **13** | **14** | **15** | **16** | **17** | **18** | **19** | **20** | **21** | **22** | |  |  |
| Albracht and Arampatzis [61] | 2013 | 1 | 1 | 1 | 1 | 0 | 1 | 1 | 0 | 1 | 1 | 1 | 0 | 1 | 1 | 0 | 1 | 1 | 1 | 0 | 0 | 1 | 0 | | 15 | M |
| Arampatzis et al. [38] | 2007 | 1 | 1 | 1 | 1 | 0 | 0 | 1 | 0 | 0 | 1 | 0 | 0 | 1 | 1 | 0 | 1 | 1 | 1 | 1 | 0 | 0 | 0 | | 12 | M |
| Arampatzis et al. [64] | 2010 | 1 | 1 | 1 | 1 | 1 | 1 | 1 | 0 | 0 | 1 | 1 | 0 | 1 | 1 | 0 | 1 | 1 | 1 | 1 | 0 | 0 | 0 | | 15 | M |
| Bohm et al. [13] | 2014 | 1 | 1 | 1 | 1 | 1 | 0 | 1 | 0 | 1 | 0 | 1 | 0 | 1 | 1 | 0 | 1 | 1 | 0 | 1 | 0 | 0 | 1 | | 14 | M |
| Bohm et al. [60] | 2021 | 1 | 1 | 1 | 1 | 1 | 1 | 1 | 0 | 1 | 0 | 1 | 0 | 1 | 1 | 0 | 1 | 1 | 1 | 1 | 0 | 1 | 1 | | 17 | L |
| Centner et al. [12] | 2019 | 1 | 1 | 1 | 1 | 0 | 1 | 1 | 1 | 0 | 0 | 1 | 0 | 1 | 1 | 0 | 1 | 0 | 0 | 1 | 0 | 1 | 1 | | 14 | M |
| Centner et al. [17] | 2023 | 1 | 1 | 1 | 1 | 1 | 1 | 1 | 0 | 0 | 0 | 1 | 1 | 1 | 1 | 0 | 1 | 0 | 1 | 1 | 0 | 1 | 0 | | 15 | M |
| Cini et al. [59] | 2024 | 0 | 1 | 1 | 1 | 1 | 1 | 1 | 1 | 1 | 0 | 1 | 0 | 1 | 1 | 0 | 1 | 1 | 0 | 1 | 0 | 0 | 1 | | 15 | M |
| Deng et al. [39] | 2020 | 1 | 1 | 1 | 1 | 1 | 1 | 1 | 0 | 1 | 0 | 1 | 0 | 1 | 1 | 1 | 1 | 1 | 1 | 1 | 0 | 0 | 1 | | 17 | L |
| Fouré et al. [76] | 2011 | 1 | 1 | 1 | 1 | 1 | 1 | 1 | 0 | 0 | 1 | 1 | 1 | 1 | 1 | 0 | 1 | 1 | 0 | 1 | 1 | 0 | 0 | | 16 | L |
| Fouré et al. [55] | 2009 | 1 | 1 | 1 | 1 | 1 | 1 | 1 | 1 | 1 | 1 | 1 | 0 | 1 | 1 | 0 | 1 | 1 | 1 | 1 | 1 | 0 | 0 | | 18 | L |
| Fouré et al. [54] | 2012 | 1 | 1 | 1 | 0 | 1 | 0 | 1 | 0 | 1 | 0 | 1 | 0 | 1 | 1 | 0 | 1 | 1 | 1 | 0 | 1 | 0 | 0 | | 13 | M |
| Fouré et al. [72] | 2013 | 1 | 1 | 1 | 1 | 1 | 1 | 1 | 0 | 1 | 0 | 1 | 0 | 1 | 1 | 0 | 1 | 1 | 0 | 1 | 1 | 0 | 0 | | 15 | M |
| Fouré et al. [65] | 2010 | 1 | 1 | 1 | 1 | 1 | 1 | 1 | 0 | 0 | 0 | 1 | 0 | 1 | 1 | 0 | 1 | 0 | 0 | 1 | 1 | 1 | 0 | | 14 | M |
| Geremia et al. [58] | 2018 | 1 | 1 | 0 | 1 | 1 | 1 | 1 | 1 | 1 | 0 | 0 | 0 | 1 | 1 | 0 | 1 | 0 | 0 | 0 | 0 | 0 | 1 | | 12 | M |
| Hansen et al. [43] | 2003 | 1 | 1 | 1 | 1 | 1 | 1 | 1 | 1 | 0 | 0 | 1 | 0 | 1 | 1 | 0 | 1 | 1 | 1 | 0 | 0 | 0 | 0 | | 14 | M |
| Hirayama et al. [66] | 2017 | 1 | 1 | 1 | 1 | 1 | 1 | 1 | 1 | 0 | 0 | 1 | 0 | 1 | 1 | 0 | 1 | 0 | 1 | 1 | 0 | 0 | 0 | | 14 | M |
| Houghton et al. [53] | 2013 | 0 | 1 | 1 | 1 | 0 | 1 | 1 | 0 | 0 | 1 | 1 | 0 | 1 | 1 | 1 | 1 | 1 | 1 | 0 | 0 | 0 | 0 | | 13 | M |
| Ikeda et al. [74] | 2024 | 1 | 1 | 1 | 1 | 1 | 1 | 1 | 1 | 1 | 1 | 1 | 0 | 1 | 1 | 0 | 1 | 1 | 1 | 1 | 0 | 0 | 1 | | 18 | L |
| Ishigaki et al. [57] | 2018 | 1 | 1 | 1 | 1 | 1 | 1 | 1 | 0 | 1 | 0 | 1 | 0 | 0 | 1 | 0 | 1 | 1 | 0 | 1 | 0 | 0 | 1 | | 14 | M |
| Jerger et al. [56] | 2022 | 1 | 1 | 1 | 1 | 1 | 1 | 1 | 1 | 1 | 1 | 1 | 1 | 1 | 1 | 0 | 1 | 1 | 1 | 1 | 1 | 0 | 1 | | 20 | L |
| Joseph et al. [36] | 2017 | 1 | 1 | 1 | 1 | 1 | 1 | 1 | 1 | 0 | 1 | 1 | 0 | 1 | 1 | 1 | 1 | 1 | 1 | 1 | 0 | 0 | 0 | | 17 | L |
| Kay et al. [41] | 2024 | 1 | 1 | 1 | 1 | 0 | 1 | 1 | 0 | 1 | 0 | 1 | 0 | 1 | 1 | 1 | 1 | 1 | 1 | 1 | 0 | 0 | 1 | | 16 | L |
| Konrad et al. [44] | 2015 | 1 | 1 | 1 | 1 | 1 | 1 | 1 | 1 | 1 | 0 | 1 | 1 | 1 | 1 | 0 | 1 | 1 | 1 | 1 | 0 | 0 | 0 | | 17 | L |
| Konrad and Tilp [51] | 2014 | 1 | 1 | 1 | 1 | 1 | 1 | 1 | 1 | 1 | 1 | 1 | 0 | 1 | 1 | 0 | 1 | 1 | 1 | 1 | 0 | 0 | 0 | | 17 | L |
| Konrad and Tilp [63] | 2014 | 1 | 1 | 1 | 1 | 1 | 1 | 1 | 1 | 0 | 1 | 1 | 0 | 1 | 1 | 0 | 1 | 1 | 1 | 1 | 0 | 0 | 0 | | 16 | L |
| Kubo et al. [73] | 2012 | 1 | 1 | 1 | 1 | 1 | 1 | 1 | 0 | 1 | 0 | 1 | 0 | 1 | 1 | 1 | 1 | 0 | 1 | 1 | 0 | 0 | 0 | | 15 | M |
| Laurent et al. [40] | 2020 | 1 | 1 | 1 | 1 | 0 | 1 | 1 | 0 | 0 | 0 | 1 | 0 | 1 | 1 | 1 | 1 | 1 | 1 | 1 | 0 | 0 | 0 | | 14 | M |
| Létocart and Grosset [11] | 2021 | 1 | 1 | 1 | 1 | 0 | 1 | 1 | 0 | 0 | 0 | 0 | 0 | 1 | 1 | 1 | 1 | 1 | 1 | 0 | 0 | 1 | 1 | | 14 | M |
| Létocart et al. [69] | 2024 | 1 | 1 | 1 | 1 | 1 | 1 | 1 | 1 | 1 | 0 | 1 | 1 | 1 | 1 | 0 | 1 | 1 | 1 | 0 | 0 | 0 | 1 | | 17 | L |
| Mahieu et al. [46] | 2007 | 1 | 1 | 1 | 1 | 1 | 1 | 1 | 1 | 1 | 0 | 1 | 0 | 1 | 1 | 1 | 1 | 1 | 1 | 1 | 0 | 0 | 0 | | 17 | L |
| Mahieu et al. [75] | 2008 | 1 | 1 | 1 | 1 | 1 | 1 | 1 | 0 | 1 | 1 | 1 | 0 | 1 | 1 | 0 | 1 | 1 | 0 | 1 | 0 | 0 | 0 | | 15 | M |
| Mahieu et al. [45] | 2009 | 1 | 1 | 1 | 1 | 1 | 1 | 1 | 0 | 1 | 0 | 1 | 0 | 1 | 1 | 1 | 1 | 1 | 1 | 1 | 0 | 0 | 0 | | 16 | L |
| Milgrom et al. [37] | 2014 | 1 | 1 | 1 | 0 | 0 | 1 | 1 | 1 | 1 | 1 | 1 | 1 | 1 | 1 | 0 | 1 | 1 | 1 | 0 | 0 | 0 | 1 | | 16 | L |
| Morrissey et al. [52] | 2011 | 1 | 1 | 1 | 1 | 1 | 1 | 1 | 0 | 1 | 0 | 1 | 0 | 1 | 1 | 1 | 1 | 0 | 1 | 1 | 0 | 0 | 1 | | 16 | L |
| Neves et al. [71] | 2020 | 1 | 1 | 1 | 1 | 1 | 0 | 1 | 1 | 1 | 1 | 1 | 1 | 1 | 1 | 0 | 1 | 0 | 1 | 1 | 0 | 0 | 1 | | 17 | L |
| Peixinho et al. [42] | 2021 | 1 | 1 | 1 | 1 | 1 | 1 | 1 | 0 | 0 | 0 | 1 | 1 | 1 | 1 | 0 | 1 | 1 | 0 | 1 | 0 | 0 | 0 | | 14 | M |
| Sanz-López et al. [79] | 2016 | 1 | 1 | 1 | 1 | 1 | 1 | 1 | 1 | 1 | 0 | 1 | 1 | 1 | 1 | 0 | 1 | 0 | 1 | 0 | 0 | 0 | 0 | | 15 | M |
| Simpson et al. [68] | 2017 | 1 | 1 | 1 | 1 | 0 | 1 | 1 | 0 | 1 | 0 | 0 | 1 | 1 | 1 | 0 | 1 | 0 | 1 | 1 | 0 | 0 | 0 | | 13 | M |
| Torres-Banduc et al. [77] | 2024 | 1 | 1 | 1 | 1 | 1 | 0 | 1 | 0 | 1 | 0 | 1 | 1 | 1 | 1 | 1 | 1 | 1 | 1 | 1 | 0 | 1 | 1 | | 18 | L |
| Tsai et al. [47] | 2024 | 1 | 1 | 1 | 1 | 1 | 0 | 1 | 0 | 0 | 0 | 1 | 1 | 1 | 0 | 1 | 1 | 0 | 1 | 1 | 0 | 1 | 1 | | 15 | M |
| Urlando and Hawkins [35] | 2007 | 1 | 1 | 1 | 1 | 0 | 1 | 1 | 0 | 1 | 0 | 0 | 0 | 1 | 1 | 0 | 1 | 1 | 1 | 0 | 0 | 0 | 0 | | 12 | M |
| Waugh et al. [62] | 2018 | 1 | 1 | 1 | 1 | 1 | 1 | 1 | 1 | 1 | 0 | 1 | 1 | 1 | 1 | 1 | 1 | 1 | 0 | 1 | 0 | 0 | 1 | | 18 | L |
| Waugh and Scott [70] | 2021 | 1 | 1 | 1 | 1 | 1 | 1 | 1 | 1 | 1 | 0 | 1 | 0 | 1 | 1 | 1 | 1 | 0 | 1 | 1 | 0 | 0 | 0 | | 16 | L |
| Werkhausen et al. [48] | 2018 | 1 | 1 | 1 | 1 | 1 | 1 | 1 | 1 | 1 | 0 | 1 | 0 | 1 | 1 | 0 | 1 | 1 | 0 | 0 | 0 | 0 | 1 | | 15 | M |
| Werkhausen et al. [78] | 2019 | 1 | 1 | 1 | 1 | 1 | 1 | 1 | 0 | 1 | 0 | 1 | 0 | 1 | 1 | 0 | 1 | 0 | 1 | 0 | 0 | 0 | 0 | | 13 | M |
| Wu et al. [67] | 2010 | 1 | 1 | 1 | 1 | 1 | 1 | 1 | 1 | 1 | 0 | 0 | 0 | 1 | 1 | 0 | 1 | 0 | 1 | 1 | 0 | 0 | 0 | | 14 | M |
| Zhang et al. [49] | 2021 | 1 | 1 | 1 | 1 | 1 | 1 | 1 | 1 | 1 | 0 | 1 | 0 | 1 | 1 | 1 | 1 | 1 | 0 | 1 | 0 | 1 | 1 | | 18 | L |
| Zhang et al. [50] | 2024 | 1 | 1 | 1 | 1 | 1 | 1 | 1 | 0 | 1 | 0 | 1 | 0 | 1 | 1 | 1 | 1 | 1 | 1 | 1 | 0 | 1 | 1 | | 18 | L |

*(1) Aim clearly described; (2) Outcomes described; (3) Subjects clearly described; (4) Interventions clearly described; (5) Distribution of confounders described; (6) Main findings clearly described; (7) Estimates of random variability in data; (8) All important adverse events reported; (9) Actual probability values reported; (10) Subjects asked representative of population; (11) Subjects used representative of population; (12) Examiners blinded; (13) Data dredging; (14) Appropriate statistical tests; (15) Internal validity; (16) Valid and reliable main outcome measures; (17) Subjects recruited from same population; (18) Subjects recruited over same time period; (19) Subjects randomised to intervention groups; (20) Intervention order randomized; (21) Adequate adjustment for confounding; (22) Sufficient power; M, moderate risk; L, low risk.
